# ILSI Europe Systematic Review: The Impact of Digestible and Nondigestible Carbohydrate Consumption for Toddlers (1–4 Years) in Relation to Health Outcomes

**DOI:** 10.1093/nutrit/nuae212

**Published:** 2025-02-05

**Authors:** Bartlomiej M Zalewski, Gisela A Weiss, Cristina Campoy, Tamás Decsi, Elisabetta Di Profio, Renaud Mestdagh, Maryam Rakhshandehroo, Hania Szajewska, Stephan Theis, Elaine E Vaughan, Elvira Verduci, Ching-Yu Chang

**Affiliations:** Department of Pediatrics, The Medical University of Warsaw, 02-091 Warsaw, Poland; Yili Innovation Center Europe, 6708 WH Wageningen, The Netherlands; Europe Research Center—National Center of Technology Innovation for Dairy (China), 6708 WH Wageningen, The Netherlands; Department of Pediatrics, School of Medicine; EURISTIKOS Excellence Centre for Pediatric Research, University of Granada, Granada, Spain; BioSanitary Institute of Granada (Ibs-Granada), Granada, Spain; CIBERESP Spanish Research Network on Epidemiology and Public Health, National Institute of Health Carlos III, Madrid, Spain; Department of Pediatrics, Faculty of Medicine, University of Pécs, Pécs, Hungary; Department of Health Science, University of Milan, Milan, Italy; Department of Dietetics and Clinical Nutrition, Maggiore della Carità Hospital, Novara, Italy; Cargill R&D Centre Europe BV, B-1800 Vilvoorde, Belgium; Danone Research and Innovation, 3584 CT Utrecht, The Netherlands; Department of Pediatrics, The Medical University of Warsaw, 02-091 Warsaw, Poland; BENEO/Suedzucker Group, 67283 Obrigheim, Germany; Sensus (Royal Cosun), 4704 RA Roosendaal, The Netherlands; Metabolic Disease Unit, Department of Pediatrics, Vittore Buzzi Hospital, University of Milan, Milan, Italy; International Life Sciences Institute, European Branch, 1200 Brussels, Belgium

**Keywords:** carbohydrate, sugar, beverage, glycemic, body composition, bowel habit

## Abstract

**Context:**

Early dietary habits play a crucial role in shaping long-term health outcomes. Understanding the effects of different carbohydrate types on physiological markers is essential for developing evidence-based nutritional guidelines for toddlers.

**Objective:**

The aim was to systematically evaluate the impact of both digestible and nondigestible carbohydrate intake during early childhood (1–4 years of age) on various health outcomes, including growth patterns, metabolic parameters, and the development of risk of cardiovascular diseases.

**Data Sources:**

PubMed, Embase, and CENTRAL databases were searched up to April 2022 to identify studies investigating carbohydrate consumption in toddlers.

**Data Extraction:**

The types of carbohydrates consumed, their sources, and their associations with growth parameters and metabolic markers were extracted. Thirty-one publications, including 18 cohort studies and 2 randomized controlled trials, were included.

**Data Analysis:**

The risk of bias was assessed using the Mixed Methods Appraisal Tool. A narrative synthesis was performed, with a visual summary table of the direction of effects.

**Conclusion:**

In toddlers, the negative impact on health risks later in life is more pronounced for digestible dietary carbohydrate intake in liquid forms, such as sugar-sweetened beverages and fruit juice, compared with solid forms. Higher nondigestible carbohydrate (dietary fiber) intake during early childhood showed a beneficial trend on later lipid profile. Further studies are required to comprehensively assess the effect of digestible and nondigestible carbohydrate intake in toddlers on cognitive and psychomotor development, infections, bowel function, and gut microbiota.

## Graphical Abstract

**Figure:**
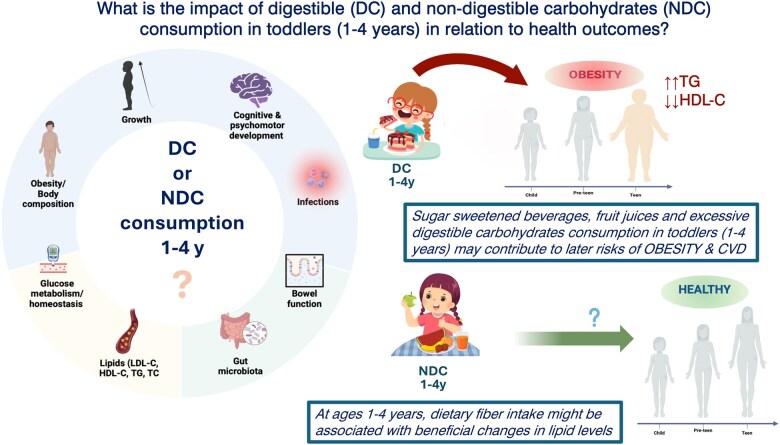

## INTRODUCTION

Despite substantial advances in understanding the role of dietary digestible and nondigestible carbohydrates (DCs and NDCs, respectively) in adult health, there is less research on their impact in young children for both short- and long-term health outcomes. In Europe, types of carbohydrates allowed in infant and follow-on formula are regulated.^1^ Upon the introduction of solids into the infants’ diet, ideally all types of carbohydrates may be introduced, mainly through fruits and vegetables.^2^^,^^3^ The European Food Safety Authority (EFSA) recommends that 45%–60% of an adult’s energy intake should come from carbohydrates, based on their effect on body weight and blood lipids, with consideration of practical factors. This recommendation has been extended to children aged 1–3 years.^3^^,^^4^ The intake of added/free sugars is recommended by the European Society for Paediatric Gastroenterology, Hepatology, and Nutrition (ESPGHAN) not to exceed 5% of energy intake in children aged 2 years and older, and to be even lower in children younger than 2 years, with the specific recommendation to replace food groups like sugar-sweetened beverages (SSBs) and fruit juices with water.^5^

However, physiological responses to different types of carbohydrates can vary. The review includes studies on various carbohydrate sources, including liquid forms such as SSBs and fruit juice, to provide a comprehensive overview of carbohydrate intake in pediatric populations. However, it is important to note that these liquid sources may present unique metabolic responses and confounding factors that limit their ability to fully represent total DC intake.

In this review, we categorize carbohydrates into 2 main types: “digestible carbohydrates” (ie, carbohydrates digested and absorbed in the human small intestine) and “dietary fiber,” which are NDCs passing to the large intestine. This definition of dietary fiber also includes lignin, as per the EFSA opinion.^4^

Nutritionally, carbohydrates can be differentiated according to their distinct effects on physiology and metabolism.^6^ Glycemic carbohydrates, which are carbohydrates digested and absorbed in the human small intestine with a subsequent increase in blood glucose, provide carbohydrates to body cells, mainly in the form of glucose. They include monosaccharides (ie, glucose, fructose), disaccharides (ie, sucrose, lactose), malto-oligosaccharides, and polysaccharides (ie, starch). In contrast, nonglycemic carbohydrates pass undigested to the large intestine and with no increase in blood glucose. These include dietary fibers and other NDCs. Nondigestible carbohydrates include non-starch polysaccharides (ie, cellulose, hemicellulose, pectin), oligosaccharides (ie, fructo-oligosaccharides [FOS], galacto-oligosaccharides [GOS]), and resistant starch. Essentially all NDCs we refer to can be characterized as dietary fiber.^4^ The EFSA established an adequate intake (AI) of dietary fiber for children aged 12 to 48 months based on the role of dietary fiber in bowel function.^4^ An AI of 10 g per day has been proposed for young children; however, many European children fall short of this target, with mean dietary fiber intakes ranging from approximately 6 to 15 g per day.^3^

Different types of carbohydrates have varying impacts on the human body. For example, some mono- and disaccharides such as glucose or sucrose provide rapid energy, while dietary fiber and NDCs play a role in improving bowel habits or glycemic response.^7^ Nondigestible carbohydrates may also play a role in shaping the intestinal microbiota, which is increasingly recognized as important for child health.^8^ Early-life nutrition and the development of gut microbiota have been linked to noncommunicable diseases, such as cardiovascular disease, obesity, type 2 diabetes, and metabolic dysfunction–associated fatty liver disease later in life.^9^^,^^10^ There is increasing evidence that intake of dietary fiber over time may play a role in the prevention of diseases such as type 2 diabetes, cardiovascular health, and colorectal cancer.^11^^,^^12^ While this evidence has largely been established in adult populations, in young children there may be immediate physical, well-being, and mental effects impacting them into adulthood.^13^ More than 10 years ago, an International Life Sciences Institute (ILSI) review on the role of DCs in infants and toddlers pointed out the limited number of carbohydrate-intake studies and the lack of information about starch intake in children younger than 4 years.^14^ Similarly, a recent ILSI workshop identified a lack of scientific data on how common foods affect glucose and insulin levels in children.^15^ Given the anticipated limited number of studies focused on this age group, we have chosen to evaluate both DC and NDC usage in toddler foods to provide a more robust evidence base for informing public health strategies, rather than diluting the findings across separate reviews. The selection of health outcomes of interest was made by experts from a cross-disciplinary team, focusing on key aspects of toddler health. Children aged 1 to 4 years old experience rapid growth, with substantial changes in both body and brain size (growth outcomes),^16^ while their immune system is still in the process of maturing (infection outcomes).^17^ These early years are also critical for establishing healthy body composition and metabolic regulation, influencing outcomes such as obesity, glucose homeostasis, and lipid profiles. This period often marks significant dietary transitions, such as the shift from breastfeeding or formula feeding to solid foods, which can substantially affect health outcomes, including bowel function, immune development, and gut microbiota composition.^18^ Both the American Academy of Pediatrics (AAP)^19^ and the World Health Organization (WHO)^20^ recommend breastfeeding alongside complementary foods for up to 2 years or longer, as desired by the mother and child. Human milk, a source of both lactose and nondigestible human milk oligosaccharides, continues to be consumed by a significant proportion of children in this age group and may influence immune system development and gut microbiota composition. The aim of this paper is to systematically review the current evidence on the impact of both DCs and NDCs in toddler nutrition, focusing on children aged 12–47 months with respect to the prespecified health outcomes of interest. Our objective was to identify existing evidence gaps and determine the additional research needed to enhance understanding of the impact of carbohydrates on toddlers.

## METHODS

This report adheres to the Preferred Reporting Items for Systematic Reviews and Meta-Analyses (PRISMA) 2020 guidelines.^21^ The review protocol was predetermined and agreed upon during the ILSI Europe Dietary Carbohydrates and Early Nutrition and Long-Term Health Task Force meetings in April/May 2022. Although the review protocol was not formally registered or published, the methods for each stage of this systematic review were predetermined and agreed upon. The inclusion criteria were established following the PI(E)COS (Population, Intervention/Exposure, Comparison, Outcomes, Study design) framework (Table 1), as follows:

**Table 1.** PI(E)COS Inclusion Criteria

Parameter	Criterion
Population	Children
Intervention/Exposure	Digestible and nondigestible carbohydrates at age 12–47 months
Comparison	Comparison of levels/different amounts of digestible and nondigestible carbohydrates; control group
Outcomes (short- and/or long-term)	1. Growth (weight, length, head circumference) 2. Infections (diagnosis of infection rather than specific measurements of infection) 3. Bowel function (stool frequency/consistency; constipation, diarrhea) 4. Cognitive and psychomotor development 5. Obesity/overweight/body composition 6. Glucose metabolism/homeostasis (including fasting plasma glucose [FPG], insulin, glycated hemoglobin [HbA1c], C-peptide, others) 7. Lipids (low-density-lipoprotein cholesterol [LDL-C]/high-density-lipoprotein cholesterol [HDL-C]/triglycerides [TG]/total cholesterol [TC]) 8. Gut (fecal) microbiota
Study design	Randomized controlled trial, prospective cohort study

- Population. Studies involving presumably healthy children from general populations were included, while those involving only children with specific diseases/conditions, such as diarrhea or obesity, were excluded. The selected age range was 12–47 months, chosen due to limited research available on this population. When studies with mixed age groups were included, at least 50% plus 1 of the participants had to be in the target age group to be considered.
- Intervention/exposure. DCs and NDCs.
- Comparison. Different amounts of DC and NDCs, or a control group.
- Outcomes. To be included in this systematic review, studies had to report at least 1 of the health outcomes (including children, adolescents, adults) listed in Table 1.
- Study design. Randomized controlled trials (RCTs) and prospective cohort studies (PCs) were included in this systematic review. Reviews focusing on studies of interest were excluded, although references were searched.

### Search Strategy

The search for eligible studies was conducted using 3 main electronic databases (PubMed, Embase, and CENTRAL) up until April 2022. The reference lists of included studies were checked. In addition, relevant systematic and non–systematic reviews that were excluded during the screening process were searched manually. Experts in the field were contacted for potential studies. No language restrictions were applied. The search strategy is provided in Table S1.

### Selection Process and Data Collection

Covidence (Melbourne, Australia), a web-based collaboration software platform, was used for the literature search, with 2 out of the 7 reviewers (B.M.Z., R.M., E.D.P., G.A.W., S.T., E.V., M.R.) independently screening titles and abstracts. For the purposes of this review, publications written in languages other than English were excluded. The full texts of potentially eligible studies were evaluated independently by 2 reviewers (B.M.Z. and 1 other author). Any disagreements regarding eligibility criteria were resolved through discussion until a consensus was reached. All reviewers agreed on the final set of included studies. The extraction process was done independently by 2 reviewers (B.M.Z. extracted data from all included papers; double extraction was carried out by 1 of the other reviewers). The following information was collected: author’s name, year of publication, study design, population, description of exposure, outcome measures and definitions, results, and information necessary to assess risk of bias.

### Quality Assessment

Assessment of the risk of bias of included studies was conducted independently by pairs of reviewers (B.M.Z. and 1 other author) using the Mixed Methods Appraisal Tool (MMAT; version 2018).^22^ In cases of disagreement among reviewers, resolution was sought through discussion with other reviewers. Interventional studies were assessed based on the criteria of appropriateness of randomization, comparability of baseline groups, outcome data completeness, blinding, and adherence to assigned intervention (questions 2.1–2.5). For observational studies, sample representativeness, outcome appropriateness and completeness, exposure measures, the presence of controlling for potential confounding factors, as well as administration of the intervention/exposure (questions 3.1–3.5) were considered (for details, see Table S2A and S2B).

### Data Analysis

Narrative synthesis was performed to summarize the findings of included studies. A meta-analysis was considered, although not conducted due to differences in exposure definitions, effect measures, and timing used across the included studies. Characteristics and details of all included studies are presented in Table 2.^23–53^ A visual summary of the direction of effects for the different outcomes assessed in the studies is presented in Table 3.^23–53^

**Table 2.** Characteristics of the Included Studies

Study (year); design	Study setting and population	Exposure/intervention and comparison	Exposure, data-collection methods	Outcome measures (of interest)	Results
Beyerlein et al (2015); prospective cohort (TEDDY study)^23^	*n* = 3358 Age: 9–48 mo Country: USA and Germany Years recruited: 2004–2010	Soluble fiber intake at 12 mo (continuous intake per gram)	3-d food record every 3 mo starting from 6 mo, until the child was 12 mo, and then every 6 mo measured by parents (reviewed by trained study personnel)	Any persistent confirmed islet autoimmunity up to a median of 5 y (presence of selected autoantibodies on ≥2 consecutive visits) Development of T1DM (American Diabetic Association 2011 criteria) up to a median of 5 y	The intake level of dietary soluble fiber at 12 mo was not associated with: Islet autoimmunity (adj. HR = 0.96; 95% CI: 0.86; 1.08; *n* = 3252 for any persistent antibody) T1DM development (adj. HR = 0.98; 95% CI: 0.80; 1.19; *n* = 3330) during 5 y of follow-up
Buyken et al (2008); prospective cohort (DONALD study)^24^	*n* = 380 (convenience sampling) Age: 2–7 y Country: Germany Years recruited: 1985–2000	Added sugar, fiber intake at 2 y Added sugars: white sugar, brown sugar, raw sugar, corn syrup, corn-syrup solids, high-fructose corn syrup, malt syrup, maple syrup, pancake syrup, fructose sweetener, liquid fructose, honey, molasses, anhydrous dextrose, and crystal dextrose, fruit syrups Fiber: dietary fiber from cereal, fruit, and vegetables	Weighed 3-d dietary records measured by parents (followed by dietitian visiting the family at home)	%BF at 2 y BMIZ at 2 y Change in %BF at 2–7 y (skinfold thicknesses) Change in BMIZ at 2–7 y Trained researchers; standardized protocol	No association between added-sugar, fiber intake at 2 y with %BF or BMIZ at 2 y (cross-sectional analyses); *n* = 380 No association between added-sugar, fiber intake at 2 y with 2–7-y change in %BF or BMIZ (longitudinal analyses); *n* = 380
Byrne et al (2018); prospective cohort (based on NOURISH RCT)^25^	*n* = 515 at 2 y, *n* = 426 at 3.7 y Age (range): 2–5 y Country: Australia Years recruited: not reported	Intake of sweet beverages at 2 and 3.7 y (grams and proportion total estimated energy) Sweet beverages: flavored milks, 100% juice, dilute juice, fruit drinks/cordials and soft drinks	24-h dietary recall at 2 and 3.7 y by dietitian	BMIZ at 2 y, 3.7 y (longitudinal analyses) Trained researchers; standardized protocol	No relationship between intake of sweet beverages and BMIZ at any age
Cantoral et al (2016); prospective cohort (convenience sample; ELEMENT study)^26^	*n* = 227 Age: Birth–14 y Country: Mexico Years recruited: 1998–2003	SSB: sum of daily intake of sodas, commercial fruit drinks and flavored water with sugar (excluding natural fruit or vegetable juices) Cumulative consumption of SSBs at 1–5 y	A semi-quantitative FFQ over last 3 mo (validated) Trained personnel administered the FFQ to the mother at each visit beginning at 12 mo	Obesity (>2 SD BMIZ) at 8–14 y Abdominal obesity at 8–14 y (waist circumference >90th percentile) Trained researchers; standardized protocol	Children in the highest tertile of cumulative SSB consumption at 1–5 y, compared with the lowest tertile, had almost 3 times the odds of obesity (adj. OR = 2.99; 95% CI: 1.27; 7.00; *n* = 227) and abdominal obesity (adj. OR = 2.70; 95% CI: 1.03; 7.03; *n* = 227) at 8–14 y
Chaidez et al (2014); prospective cohort (Supplemental Nutrition Program for Women, Infants, and Children [WIC] study)^27^	*n* = 67 (convenience sampling) Age: 12–24 mo at baseline; follow-up ∼6 mo later Country: USA Years recruited: not reported	High added sugars at baseline (as % of kcal intake) High SSB at baseline (did not include 100% fruit juice) Added sugars not further defined High (every value above the median)	Four 24-h dietary recalls at baseline and 6-mo follow-up (self-reported by mothers)	6-mo change in WHZ, WAZ, BMIZ Average age of children at follow-up, 27.7 mo Trained researchers; standardized protocol	High intake of SSBs at baseline was associated at 6-mo follow up with: Increase in WHZ; adj. β = 0.46; *P* < .05; *n* = 52 Increase in BMIZ; adj. β = 0.47; *P* = .07; *n* = 51 No change in WAZ; adj. β = 0.13; *P* = .35; *n* = 56 High intake of added sugars at baseline was not associated with any change in measured anthropometrical outcomes at 6-mo follow-up
Cowin et al (2001); prospective cohort (randomly selected group) (ALSPAC)^28^	*n* = 389 Age: 18 mo (at baseline); 31 mo (follow-up) Country: UK Years recruited: 1991–1992	Total carbohydrate, starch, sugar, fiber (non-starch polysaccharides) intake at 18 mo	3-d unweighted dietary record reported by parent (and discussed with nutritionist) at 18 mo	TC, HDL-C, LDL-C, TC:HDL-C (nonfasting), and height and BMI at 31 mo Trained researchers; standardized protocol	Boys (*n* = 133–214) Univariate analysis: No report on associations (or lack of) with BMI Height at 31 mo was negatively associated with the energy-adjusted intakes of total carbohydrate (*r* = −0.14, *P* = .02) and starch (*r* = −0.12; *P* = .01) at 18 mo TC was negatively associated with energy-adjusted intakes of total carbohydrate (*r* = −0.17; *P* = .01) and positively with energy-adjusted intakes of sugar (*r* = 0.15; *P* = .03) HDL-C and LDL-C levels at 31 mo not associated with energy-adjusted intakes of starch or sugar at 18 mo TC:HDL-C ratio was negatively associated with energy-adjusted total carbohydrate intake at 18 mo (*r* = −0.16; *P* = .04), but not associated with energy-adjusted intakes of starch or sugar No evidence that fiber intake has any association with blood lipid concentrations Multivariate analysis: No significant associations of sugar intake with blood lipid concentrations Girls (*n* = 109–175) Univariate analysis: No report on associations (or lack of) with height, BMI, and nutrients of interest TC, HDL-C, LDL-C, TC:HDL-C ratio at 31 mo was not associated with starch, sugar intake at 18 mo No evidence that fiber intake has any association with blood lipid concentrations Multivariate analysis: TC:HDL-C ratio non-significantly positively associated with sugar intake at 18 mo
DeBoer et al (2013); prospective cohort (Early Childhood Longitudinal Survey Birth Cohort [ECLS-B])^29^	*n* = 9600 Age: 2–5 y Country: USA Years recruited: children born in 2001	SSB intake at 2 y SSBs: soda pop, sports drinks or fruit drinks that are not 100% fruit juice Regular drinker: SSBs at either meals/snacks Infrequent/nondrinker: no SSB consumption at meals/snacks	Parents were interviewed in their home by trained assessors at 2 y, 4 y	BMIZ at 2 y Overweight/obesity at 2 y BMI ≥85th percentile, defined as overweight (CDC 2000) BMI ≥95th percentile, defined as obesity (CDC 2000) Trained researchers; standardized protocol	Higher rates of SSB consumption (≥1 serving of SSB/d) at 2 y were not associated with overweight (adj. OR = 1.0; 95% CI: 0.80; 1.24) or obesity (adj. OR = 1.01; 95% CI: 0.72; 1.42) at 2 y (*n* = 7600) Higher SSB consumption (≥1 serving of SSB/d) at 2 y resulted in similar adj. mean BMIZ at 2 y
Dubois et al (2007); prospective cohort (Longitudinal Study of Child Development)^30^	*n* = 1944 Age (range): 2.5–4.5y Country: Canada Years recruited: 1998–2002	Frequency of SSB consumption between meals (regular consumers ≥4–6 times/wk) SSB: a drink that has added sugar, eg, regular or non-diet carbonated drinks and fruit-flavored drinks (eg, fruit punch and orange drinks) Pure fruit juices, eg, 100% apple or orange juice, were excluded	FFQ completed by the mother at 2.5, 3.5, and 4.5 y	Obesity at 4.5 y Obesity (BMI ≥95th percentile; CDC 2000) Trained researchers; standardized protocol	Increased odds of obesity at 4.5 y in regular consumers at age 2.5, 3.5, and 4.5 y compared with non-consumers (adj. OR = 2.36; 95% CI: 1.03; 5.39; *n* = 1944)
Garden et al (2011); prospective cohort (based on RCT CAPS study)^31^	*n* = 362 Age (range): 1–18 mo (8 y at outcome) Country: Australia Years recruited: 1997–1999	Carbohydrate intake at 18 mo Percentage of total energy from carbohydrates at 18 mo Non-milk beverages intake at 18 mo (juice, cordials, fruit drinks, and soft drinks) Sweetened drinks intake at 18 mo (cordials, fruit drinks, and soft drinks)	Weighed 3-d dietary records at 18 mo checked by dietitian	BMI at 8 y Waist circumference at 8 y Anthropometric measurements made by trained researchers; standardized protocol	Carbohydrate intake as well as % of total energy from carbohydrates at 18 mo did not predict BMI at 8 y (adj. β = 0.37; 95% CI: −0.49; 1.23; *P* = .40; adj. β = −0.01; 95% CI: 0.05; 0.03; *P* = .62 respectively; *n* = 362) Absolute carbohydrate intake at 18 mo and associations with waist circumference at 8 y did not remain significant after adjustment (β = 2.91; 95% CI: 0.59; 5.24; *P* = .01; adj. β = −0.29; 95% CI: −4.53; 3.94; *P* = .89; *n* = 362) The % of total energy from carbohydrates at 18 mo was not associated with waist circumference at 8 y in unadj/adj. analyses Non-milk beverages or sweetened drinks intakes at 18 mo were not associated with BMI/waist circumference at 8 y in unadj/adj. analyses
Herbst et al (2011); prospective cohort (DONALD study)^32^	*n* = 216 (convenience sampling) Age: 3 mo to 7 y Country: Germany Years recruited: 1990–2010	Different added-sugar types intake assessed at 1 y and as a change within 1–2 y Added sugar: white sugar, brown sugar, raw sugar, corn syrup, corn-syrup solids, high-fructose corn syrup, malt syrup, maple syrup, pancake syrup, fructose sweetener, liquid fructose, honey, molasses, anhydrous dextrose, crystal dextrose, and fruit syrups Added sugar from beverages and sweets: added sugar from regular and diet soft drinks and fruit juices; candy, chocolate, jam, and ice cream	3-d weighed dietary records checked by dietitian	BMIZ and %BF (skinfold thickness measurements) at age 7 y Anthropometric measurements made by trained researchers; standardized protocol	Total added-sugar intake: Higher intake at 1 y related to lower BMIZ at 7 y (adj. β = −0.12 ± 0.06 SD; *P* = .04; *n* = 216) Higher intake in the second year of life tended to be related to a higher BMIZ at age 7 y (adj. β = 0.07 ± 0.04 SD; *P* = 0.09; *n* = 216) Intake at 1 y or change in intake in the second year of life were not related to %BF at 7 y (adj. β = −0.01 ± 0.02; *P* = .4; and adj. β ± SD = 0.00 ± 0.01; *P* = 0.8, respectively; *n* = 216) Added sugar from beverages and sweets: Higher intake at 1 y related to lower BMIZ at 7 y (adj. β = −0.25 ± 0.10; *P* = .02; *n* = 216) Higher intake in the second year of life was not related to a higher BMIZ at 7 y (adj. β = 0.05 ± 0.06; *P* = .4; *n* = 216) Intake at 1 y or change in intake between 1 and 2 y was not related to %BF at 7 y (adj. β = −0.04 ± 0.03; *P* = .1; and −0.00 ± 0.02; *P* = .8, respectively; *n* = 216)
Huus et al (2008); prospective cohort (ABIS study)^33^	*n* = 8764 Age (range): 2.5–5 y Country: Sweden Years recruited: born between 1997 and 1999	Candy intake at 2.5 y (daily, 3–5 times/wk, 1–2 times/wk, <1 time/wk, never)	FFQ completed by the parents at 2.5 y	Overweight/obesity at 5 y (International Obesity Task Force definition) Children’s weight and height reported by parents (reports at 5 y were validated against clinic records)	No association between candy intake at 2.5 y and overweight/obesity at 5 y (adj. analyses, *n* = 7356)
Jardí et al (2019); prospective cohort (DeFensas study)^34^	*n* = 81 Age: birth to 30 mo Country: Spain Years recruited: not stated	Free sugars; natural sugars; fiber; starches intake at 12 mo Fiber intake (not defined) at 12 mo Classification of simple carbohydrates: natural sugars (in whole fruits, vegetables, milk and cereals [rice, bread, pasta, flour]); free sugars (in sweetened dairy desserts, sugary drinks [fresh and processed juices, soft drinks], sweetened cereals [sweetened breakfast cereals, biscuits, baked goods], and chocolate, sugar and honey)	A 24-h dietary recall at 12 mo through standardized interviews with parents held by 2 trained dietitians	WHZ at 30 mo (without vs with excess weight) Excess weight defined as overweight (+1 < WHZ ≤ +2) or obesity (WHZ > +2) at 30 mo Anthropometric measurements made by trained researchers; standardized protocol	The higher intake of free sugars at 12 mo was associated with an increased risk of excess weight at 30 mo (adj. OR = 1.13; 95% CI: 1.03; 1.25; *P* = .01; *n* = 81) Consumption of natural sugars, starches, and fiber at 12 mo was not associated with an increased probability of excess weight at 30 mo: Natural sugars (adj. OR = 1.14; 95% CI: 0.82; 1.60; *P* = .45; *n* = 81) Starches (adj. OR = 1.10; 95% CI: 0.96; 1.26; *P* = .15; *n* = 81) Fiber (adj. OR = 0.90; 95% CI: 0.78; 1.05; *P* = .18; *n* = 81) Consumption of free sugars, natural sugars, starches, and fiber at 12 mo was not associated with an increased probability of excess weight at the same age (cross-sectional analyses)
Kiefte-de Jong et al (2013); prospective cohort (Generation R Study)^35^	*n* = 2420 Age: 14–48 mo Country: The Netherlands Years recruited: 2002–2006	Dietary fiber; energy % from carbohydrates (polysaccharides and mono- and disaccharides) intake at 14 mo; mean (SD)	Validated semi-quantitative FFQ obtained by primary caregiver	Information on constipation by parent-derived questionnaires at 24, 36, 48 mo	Children at 24, 36, and 48 mo with vs without constipation (unadj): Dietary fiber intake: 17 g/d (SD ±9 g/d) vs 18 g/d (SD ±9 g/d); *P* > .5 for between-group difference Lower energy % from polysaccharides and higher energy % from mono- and disaccharides consumed by children with constipation vs without it (24% vs 23%, *P* = .06, and 35% vs 34%, *P* = .12, at the age of 48 mo, but no difference at 24 and 36 mo)
Leermakers et al (2015a); prospective cohort (Generation R Study)^37^	*n* = 2045 Age: 13 mo–6 y Country: The Netherlands Years recruited: 2002–2006	SCB intake at 13 mo SCBs: (SSBs including 100% fruit juices and fruit juices with added sugar)	Validated semi-quantitative FFQ, obtained by primary caregiver at 13 mo	Lipids at 6 y Anthropometric measurements made by trained researchers; standardized protocol	Children with higher SCB intake: TG = +0.12 SD; 95% CI: −0.01; 0.25; HDL-C: −0.12 SD; 95% CI: −0.25; 0.01; for highest vs lowest tertile of SCB intake There were no significant associations of SCB intake with TC, LDL-C
Leermakers et al (2015b); prospective cohort (Generation R Study)^36^	*n* = 2371 Age: 13 mo–6 y Country: The Netherlands Years recruited: 2002–2006	SCB intake at 13 mo (low: 3 servings/wk; medium: 8 servings/wk; high: 15 servings/wk) 1 serving = 150 mL SCBs: SSBs including fruit juices, fruit concentrates, lemonades, soft drinks, sports drinks	Validated semi-quantitative FFQ, obtained by primary caregiver	BMIZ, WAZ, HAZ difference between 13–48 mo and at 6 y Difference in %BF (DXA scan) between 13 mo and 6 y Difference in android:gynoid fat ratio between 13 mo at 6 y Anthropometric measurements made by trained researchers; standardized protocol	In girls, higher SCB intake at 13 mo was significantly associated with higher BMIZ at 2 y (adj. BMIZ change = 0.15; 95% CI: 0.01; 0.30); 3 y (adj. BMIZ change = 0.14; 95% CI: 0.01; 0.27); 4 y (adj. BMIZ change = 0.13; 95% CI: 0.01; 0.25) and 6 y (adj. BMIZ change = 0.11; +0.00; 0.23); and tended to be associated with higher WAZ and HAZ (*P* = .09 and .19, respectively) In boys, there was no association with BMIZ, WAZ at 6 y, but boys with high SCB intake at 13 mo were taller at age 6 y (adj. HAZ change = 0.14; 95% CI: +0.00; 0.27); *n* = 2371 boys and girls %BF at 6 y was not associated with SCB intake at 13 mo (boys and girls) Android:gynoid ratio at 6 y (body composition) was not associated with SCB intake at 13 mo in boys; however, a trend towards higher ratio was observed in girls (*P* = .09; *n* = 2001)
Lim et al (2008); prospective cohort (Detroit Dental Health Project)^38^	*n* = 365 (baseline relationships); *n* = 275 (non-overweight children at baseline; longitudinal) Age: children aged 3–4 y at entry, 66.8% of whole population (3 y: *n* = 119; 4 y: *n* = 126; 5 y: *n* = 120) Country: USA Years recruited: 2002–2003, with follow-up at 2 y	SSB defined by combining soda and fruit drinks Soda (excluding diet soda), fruit drinks (including Kool-Aid (Kraft Heinz, US), Gatorade (PepsiCo, US), Sunny Delight (Sunny Delight Beverages, US), Hi-C (Minute Maid, US), Hawaiian Punch (Keurig Dr Pepper, US), Ocean Spray (Ocean Spray, US); although excluding 100% fruit juice)	FFQ (Block Kids Food FFQ) obtained by trained interviewers at baseline	BMIZ at baseline (cross-sectional analyses) Becoming overweight or obese after 2 y (BMI ≥85th percentile: overweight; BMI ≥95th percentile: obesity) (CDC 2000) All participants weighted in accordance with standard procedures	Baseline intakes of soda and all SSBs (but not fruit drinks) were positively and significantly associated with baseline BMIZ: adj. β = 0.02; SE: 0.01; *P* = .02; for all SSBs Baseline intakes of fruit drinks and all SSBs (but not soda) were positively associated with odds ratios in the event that non-overweight children at baseline became overweight or obese after 2 y of follow-up: adj. OR = 1.04; 95% CI: 1.01; 1.07 for all SSBs; *n* = 365
Newby et al (2003); prospective cohort (North Dakota WIC Program)^39^	*n* = 1379 Age: 2–5 y (mean 2.9 y) Country: USA Years recruited: 1995–1998, with follow-up 6–12 mo	Fiber (g/d) at baseline and during follow-up after 6–12 mo	Semi-quantitative FFQ (reported as validated) obtained from mother at 2.9 y	Annual change in weight (kg/y) Measurements done by trained personnel	No significant relation between total intake of fiber (g/d) at 2.9 y and annual change in weight: adj. β = 0.01; SE: 0.02; *P* = .53; *n* = 1379
Newby et al (2004); prospective cohort (North Dakota WIC Program)^40^	*n* = 1345 Age: 2–5 y (mean: 2.9 y) Country: USA Years recruited: 1995–1998	Fruit juice, soda, diet soda, fruit drinks (servings/d); at baseline and during follow-up after 6–12 mo Fruit juice (orange juice, apple juice, and other 100% fruit juices); fruit drink (lemonade and fruit punch); soda (any non-diet soda); diet soda (all no- or low-calorie soda)	Semi-quantitative FFQ (reported as validated) at 2.9 y	Weight change/y BMI change/y Weight change (difference in weight at visit 1 and visit 2 divided by the time interval in months, then multiplied by 12); measured using standardized protocols	Annual weight change was not significantly related to intakes in ounces/d (*n* = 1345): - Fruit juice (adj. β = 0.01 lb/y; 95% CI: −0.01; 0.20); *P* = .28 - Fruit drinks (adj. β = −0.03 lb/y; 95% CI: −0.07; 0.01); *P* = .28 - Soda (adj. β = −0.00 lb/y; 95% CI: −0.08; 0.08); *P* = 0.95 - Diet soda (adj. β = 0.01 lb/y; 95% CI: −0.11; 0.13); *P* = .82 All results remained null when the outcome was BMI change/y
Nguyen et al (2020); prospective cohort (Generation R Study)^41^	*n* = 3573 Age: 13 mo–6 y Country: The Netherlands Years recruited: 2002–2006	Total carbohydrate; monosaccharides and disaccharides; polysaccharides intakes assessed at 12.9 mo	Semi-quantitative FFQ, obtained by primary caregiver, validated against three 24-h recalls	Body composition (FMI SDS; FFMI SDS at 6 and 10 y) BMIZ (8 different time points of measurements between 1 and 10 y); blood lipids and insulin levels (at 6 and 10 y) Anthropometric measurements made by trained researchers; standardized protocol	A higher total carbohydrate intake at 1 y was associated with higher levels of TG at 10 y (adj. β = +0.02 SDS per 10 g/d higher carbohydrate intake; 95% CI: 0.01; 0.04; *n* = 2548) No association of total carbohydrate intake at 1 y with BMIZ, FMI-SDS, FFMI-SDS, TC, HDL-C, LDL-C, insulin levels at 10 y Higher mono- and disaccharide intake at 1 y was associated with higher TG (adj. β = 0.02 SDS; 95% CI: 0.01; 0.04; *n* = 2548) and associated with lower HDL-C (adj. β = −0.03 SDS; 95% CI: −0.04; −0.01; *n* = 2554) at 10 y No association of mono- and disaccharide intake at 1 y and BMIZ, FMI-SDS, FFMI-SDS, TC, LDL-C, insulin at 10 y Polysaccharide intake at 1 y not associated with any of the outcomes at 10 y
Quah et al (2019); prospective cohort (GUSTO study)^42^	*n* = 555 Age: 18 mo–6 y Country: Singapore Years recruited: 2009–2010	SSB intake at 18 mo SSBs: carbonated, non-carbonated drinks, and only repackaged fruit juices with added sugar as well as malted drinks, cultured milk drinks, soya-based drinks, and traditional drinks (ie, barley water, chrysanthemum tea, herbal tea)	The child’s primary caregiver at 18 mo (self-administered 94-item FFQ)	At 6 y, BMIZ and SSF At 6 y, overweight and obesity combined (>1 SD in accordance with WHO) Anthropometric measurements and 4 skinfold thicknesses (triceps, biceps, subscapular and supra-iliac) were obtained in duplicate by trained staff; standardized protocols	SSB intakes at 18 mo (modeled as 100-mL/d increments) were associated only with higher risk of overweight/obesity at 6 y (adj. RR = 1.09; 95% CI: 1.02; 1.64; *n* = 555), but not with BMIZ scores or SSF No significant association between the medium/high SSB intake compared with the lowest intake at 18 mo for BMIZ, SSF, risk of overweight/obesity at age 6 y outcomes (*P* for trend = .68; .85; .20, respectively)
Scaglioni et al (2000); prospective cohort^43^	*n* = 171 (147 at 5 y) Age: birth–5 y Country: Italy Years recruited: 1991	Carbohydrates intake at 1 y	116-item FFQ obtained by trained dietitian at 1 y	Overweight status at 5 y (≤90th centile—defined as without overweight—vs >90th centile of Rolland-Cachera curves—defined as overweight) Measurements by trained staff; standardized protocols	In multiple logistic analysis there was no difference in regard to carbohydrates intake at 1 y in children with or without overweight at 5 y (data not shown); *n* = 147
Shefferly et al (2016); prospective cohort (Early Childhood Longitudinal Study, Birth Cohort [ECLS-B])^44^	*n* = 8950 Age (range): 2–5 y Country: USA Years recruited: born 2001	Fruit juice consumption at 2 y and 4 y: at 2 y (drinking juice at/between meals categorized as regular drinkers); at 4 y (<1 serving/d; ≥1 serving/d) Fruit juice: 100% orange/apple/grape fruit juice	Computer-assisted interview with primary caregiver	BMIZ: between 2 and 4 y WAZ: 2–4 y HAZ: between 2 and 4 y Risk of overweight/obesity between 2 and 4 y Cross-sectional analyses of 100% fruit juice consumption and BMIZ at the same age, as well as overweight/obesity status at the same time Height and weight measurements obtained by trained researchers using standardized methods	Regular vs infrequent fruit juice drinkers at 2 y: increased BMIZ difference between 2 and 4 y (*P* **= +**0.00); decreased HAZ between 2 and 4 y (*P* = +0.00) ; but not WAZ between 2 and 4 y (*P* = .06); *n* = 8950 Regular fruit juice drinkers at 2 y had increased odds of overweight between 2 and 4 y (adj. OR: 1.30; 95% CI: 1.06; 1.59; *P* = .01), but not obesity between 2 and 4 y (adj. OR: 1.30; 95% CI: 0.93–1.83; *P* = .13); *n* = 8950 Cross-sectional analyses: Lower BMIZ at 2 y for children who consistently drank juice (*P* < .05) No difference in overweight/obesity
Sonneville et al (2015); prospective cohort (Project Viva)^45^	*n* = 1163 Age: 1–8 y Country: USA Years recruited: 1999–2002	Daily fruit juice intake (orange juice and other 100% juice) at 1 y Amounts/d: small (0 oz, 1–7 oz); medium (8–15 oz); large (≥16 oz) Not clear whether the juice reported was 100% juice	Semi-quantitative FFQ (not validated for juice intake) at 1 y	WLZ at 1 y BMIZ during early (median: 3.1 y) and mid-childhood (median: 7.7 y) using standardized protocols	Fruit juice intake was not associated with WLZ at 1 y (*P* = .52; *n* = 1038) Children who drank medium and large amounts of juice at 1 y had higher BMIZ during: - Early childhood (medium amounts: adj. β = 0.16; 95% CI: 0.01; 0.32; large amounts: adj. β = 0.28; 95% CI: 0.01; 0.56) - Mid-childhood (medium amounts: adj β = 0.23; 95% CI: 0.07; 0.39; large amounts: adj. β = 0.36; 95% CI: 0.08; 0.64)
Tappin et al (2020); prospective cohort (ALSPAC study)^46^	*n* = 13 954 (at 1 y) Age (range): 4 wk–10 y Country: England Years recruited: 1991–1992	Fiber intake at 2 y	FFQ at 2 y (responses to foods containing fiber), by main carer	Questionnaire on stool frequency and consistency at 2.5 y Presence of child constipation during last 12 mo (parental reports) at ages 4.5, 5.5, 6.5, 7.5, 9.5 y Results are presented as a change in multinomial odds for a 1-SD change in the continuous measure of fiber intake	Fiber intake at 2 y and hard stool frequency at 2.5 y (no hard stools as reference); *n* = 6141–9452: Hard stool frequency “sometimes”: adj. OR = 0.85; 95% CI: 0.78; 0.93 Hard stool frequency “usually”: adj. OR = 0.82; 95% CI: 0.74; 0.90 Change in fiber intake at 2 y and longitudinal patterns of constipation at 4–10 y; *n* = 8401 (low risk of constipation as reference): Late (≥6 y) childhood constipation: adj. OR = 1.07; 95% CI: 0.92; 1.25; Early (<6 y) childhood constipation: adj. OR = 0.97; 95% CI: 0.83; 1.13 Persistent constipation: adj. OR = 0.75; 95% CI: 0.64; 0.87
Taylor et al (2016); prospective cohort (ALSPAC study)^47^	*n* = 9544 Age (range): 24–42 mo Country: UK Year recruited: 1991–1992	Fiber intake at 38 mo 0.7 < Tertile 1 < 7.4 < tertile 2 < 9.7 tertile 3 < 22.5 (g/d)	FFQ at 38 mo (self-completed by the parents)	Stool hardness reported by parents at 42 mo (as hard stools usually, sometimes, never)	Tertiles of fiber intake were associated with stool hardness at 42 mo. Children in tertile 1 were almost twice as likely to “usually” have hard stools compared with children from tertile 3 (highest fiber intake, as reference): For tertile 1: OR = 1.87; 95% CI: 1.61; 2.16; *P* < +0.00 For tertile 2: OR = 1.47; 95% CI: 1.27; 1.71; *P* < +0.00 *n* = 8899
Van Gijssel et al (2016); prospective cohort (Generation R Study)^48^	*n* = 2032 (1314–1995 in analyses) Age: ∼13 mo–6 y Country: The Netherlands Years recruited: 2002–2006	Dietary fiber intake, energy-adjusted (per 1 g/d) Dietary fiber (plant cell wall components that are not digestible by human digestive enzymes, including, eg, lignin, cellulose, hemicellulose, and pectin)	211-item semi-quantitative validated FFQ; obtained by primary caregiver at median age of 12.9 mo	Lipids (HDL-C; TG) at 6 y %BF SDS at 6 y Insulin SDS at 6 y	Higher adj. dietary fiber intake at 12.9 mo was associated with: Higher HDL-C SDS (adj. β = 0.03; 95% CI: 0.01; 0.04; *n* = 1385); lower TG SDS (adj. β = −0.02; 95% CI: −0.04; −0.00; *n* = 1383) Higher adj. dietary fiber intake at 12.9 mo was not significantly associated with: %BF SDS at 6 y (adj. β = −0.00; 95% CI: −0.02; 0.01; *n* = 1988); insulin SDS at 6 y (adj. β = −0.00; 95% CI: −0.02; 0.02; *n* = 1380)
Warner et al (2006); prospective cohort (Center for the Health Assessment of Mothers and Children of Salinas(CHAMACOS) study)^49^	*n* = 354 Age: Birth–2 y Country: USA Years recruited: 2002–2003	Daily soda (“Coca Cola or Sprite-like”) consumption at 24 mo (none, ≥1; <1) Sweets (candy, cookies, cake, ice cream, or pan dulce) consumption at 24 mo	Mothers interviewed at 24 mo with specific questions regarding children soda and sweets consumption	Obesity (≥95th BMI percentile [CDC 2000]) at 2 y Measurements by trained staff; standardized protocols	≥1 Soda consumption/d vs no consumption at 2 y increased odds of overweight at 2 y (adj. OR = 3.39; 95% CI: 1.43; 8.07; *n* = 354) <1 Soda consumption/d vs no consumption at 2 y did not increase odds of overweight at 2 y (adj. OR = 0.97; 95% CI: 0.47; 1.99; *n* = 354) 1 or >1 sweets consumption/d did not increase odds of overweight at 2 y (OR = 0.77; 95% CI: 0.39; 1.51; and OR = 0.96; 95% CI: 0.40; 2.16, respectively) when compared with <1 sweet/d at 2 y (cross-sectional analyses); *n* = 354
Williams and Strobino (2008); prospective cohort (Healthy Start Project)^50^	*n* = 519 Age: 3–4 y (mean: 3.9 y); followed-up to 7–10 y Country: USA Years recruited: 1995–1996	Dietary sucrose at 3–4 y (mean: 3.9 y) Dietary fiber at 3–4 y (mean: 3.9 y)	24-h dietary recall obtained by direct observation of the children as they ate their meals and snacks at the centers, with plate waste measurement and telephone interviews with the primary adult	BMI at 7–10 y (mean: 8.2 y) Non-fasting TC, HDL-C, TG at 7–10 y (mean: 8.2 y) Measurements by trained staff; standardized protocols	Sucrose intake at 3–4 y was significantly inversely associated with BMI at 7–10 y (adj. β = −0.10; *P* < .05; *n* = 252) but was not associated with lipids (TC, HDL-C, TG) Dietary fiber intake at 3–4 y was significantly, inversely associated with TC at 7–10 y (adj. β = −0.14; *P* < .05; *n* = 252) but was not associated with BMI, HDL-C, TG
Wu et al (2021); prospective cohort (Project Viva)^51^	*n* = 783 Age: 1–13 y Country: USA Years recruited: 1999–2002	Fruit juice intake at ∼1 y (per 1 serving; no definition) 1 serving = 4 oz or 120 mL	FFQ (reported by mothers) at ∼1 y of the average quantity of infant fruit juice consumption in an average day over the past month	Abdominal adiposity: VAT, SAAT, and TAAT assessed by DXA scans at 8 and 13 y	Higher fruit juice intake at 1 y was associated with persistently greater: VAT area SDS at 8 and 13 y (adj. β = +0.08 SD units per 1 serving/d; 95% CI: 0.03; 0.13; *P* = +0.00; *n* = 636) SAAT (adj. β = +0.05 SD units per 1 serving/d; 95% CI: 0.00; 0.09; *P* = .03; *n* = 636) and TAAT (adj. β = +0.05 SD units per 1 serving/d; 95% CI: 0.01; 0.10; *P* = .01; *n* = 636) area SDS at 8 and 13 y
Nakamura et al (2006); RCT^52^	*n* = 150 (urban slum community) Age: 25–59 mo (mean: 46 mo) Country: Bangladesh Years recruited: 2004–2005	75 children in the FOS group received 50 mL of isotonic solution with 2 g of FOS/d for 6 mo 75 children in the placebo group received same solution with 1 g of added glucose/d for 6 mo	—	Primary outcomes: improvement in body weight gain every second day during 6 mo; reduction in diarrhea episodes Secondary outcomes: diarrhea duration per 6-mo period, duration of single diarrheal episode, number of defecations/d per diarrhea episode	Body-weight gain (no significant difference between groups; *P* value not reported) Number of diarrheal episodes per 6-mo period non-significantly different; *P* = .10 Secondary: A significant reduction in duration of diarrhea per single episode as well as reduction in cumulative number of diarrhea days per 6-mo period in the FOS vs placebo group (*P* = .01 and *P* = +0.00, respectively) Number of defecations/d per diarrhea episode not different (*P* = .1)
Waligora-Dupriet et al (2007); RCT^53^	*n* = 20 (out of 35) Age (range): 7–19 mo Country: France Years recruited: not specified	Run-in (8 d), followed by 21 d supplementation of 2 g/d FOS (oligofructose) or placebo (maltodextrin) in 1 dose added to foods or drinks (day 28), and 15-d run-out period (day 42)	—	Bacterial counts in stools via culturing (mean ± SD log_10_ CFU/g) Stools for bacteriological analysis were collected at 4 periods: (1) at the end of the observation period (day [D] 8), (2) in the middle of the supplementation period (D18 ± 2 d), (3) at the end of the supplementation period (D28 ± 2 d), (4) at the end of the post-supplementation period (D42) Gastrointestinal tolerance symptoms and well-being: (1) number and stool type; (2) bloating, pain, flatus, regurgitation; vomiting, less appetite, feeding refusal, fever; (3) use of medication (antibiotics), occurrence of illnesses	In the FOS group, in 10 colonized children vs 10 receiving placebo, at D28: - Bifidobacteria levels non-significantly increased by a mean 9.5 log_10_ CFU/g ± SD 0.8 vs 9.0 log_10_ CFU/g ± SD 0.7; *P* ≤ .1 - Clostridia levels non-significantly decreased by 7.2 ± SD 1.8 vs. 7.2 ± SD 1.5; *P* ≤ .1; during the supplementation period only Tolerance and well-being: - Frequency and consistency of the stools similar between FOS and placebo (*P* > .05) In the FOS group. less (reported) diarrhea (*P* < .001) vs control - The number of infectious diseases requiring antibiotic treatment, diarrhea was significantly lower in the FOS group as compared with the control group (*P* < .001)

Abbreviations: adj., adjusted; ALSPAC, Avon Longitudinal Study of Parents and Children; %BF, body fat percentage; BMI, body mass index; BMIZ, BMI *z* score; CDC, Centers for Disease Control and Prevention; CFU, colony-forming units; DONALD, Dortmund Nutritional and Anthropometric Longitudinally Designed Study; DXA, dual-energy X-ray absorptiometry; ELEMENT, Early Life Exposure in Mexico to Environmental Toxicants project; FFMI, fat-free mass index; FFQ, food-frequency questionnaire; FMI, fat mass index; FOS, fructo-oligosaccharides; HDL-C, high-density-lipoprotein cholesterol; HR, hazard ratio; LDL-C, low-density-lipoprotein cholesterol; OR, odds ratio; RCT, randomized controlled trial; RR, relative risk; SAAT, subcutaneous abdominal adipose tissue; SCB, sugar-containing beverage; SDS, sex- and age-independent SD scores; SSB, sugar sweetened beverage; SSF, sum of skinfolds; T1DM, type 1 diabetes mellitus; TAAT, total abdominal adipose tissue; TEDDY, The Environmental Determinants of Diabetes in the Young study; TC, total cholesterol; TG, triglycerides; WAZ, weight-for-age *z* score; WHZ, weight-for-height *z* score; WLZ, weight-for-length *z* score; unadj, unadjusted; VAT, visceral adipose tissue; WHO, World Health Organization.

**Table 3.** Direction of Effects Assessed in the Included Studies

Exposure and study reference	Outcomes of interest^a^							Risk of bias^b^
	Growth	Infection	Bowel function	Obesity, overweight, body composition	Glucose metabolism and homeostasis	Lipids	Microbiota	
**Digestible carbohydrates**								
**Glycemic carbohydrates**								
**Chaidez et al** (2014)^27^ (WIC)	Added sugars at 12-24 mo: < > WAZ after 6 mo	—	—	Added sugars at 12-24 mo: < > WHZ after 6 mo < > BMZ after 6 mo	—	—	—	Some
**Cowin et al** (2001)^28^ (ALSPAC)	Total carbohydrates at 18 mo: ▲Height (boys) < > Height (girls) at 31 mo Starch at 18 mo: ▲Height (boys) < > Height (girls) at 31 mo Sugars at 18 mo: < > Height at 31 mo	—	—	Total carbohydrates, starch, sugars at 18 mo: < > BMI at 31 mo	—	Total carbohydrates at 18 mo: ▼TC (boys) ▼TC:HDL-C (boys) < > TC (girls), HDL-C, LDL-C, TC:HDL-C (girls) at 31 mo Starch at 18 mo: < > TC, LDL-C, HDL-C, TC:HDL-C at 31 mo Sugar at 18 mo: ▲TC (boys) < > TC (girls), HDL-C, LDL-C, TC:HDL-C (at 31 mo; univariate) Sugar at 18 mo: < > TC, LDL-C, HDL-C, TC:HDL-C (boys), 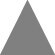 TC:HDL-C (girls) (at 31 mo; multivariate) Starch, total carbohydrates intake at 18 mo: < > Lipids (at 31 mo; multivariate)	—	Low
**DONALD study:**								
Buyken et al (2008)^24^	—	—	—	Added sugars at 2 y: < > %BF, BMIZ (at 2 y; 2-7 y change)	—	—	—	Some
Herbst et al (2011)^32^	—	—	—	Added sugars: < > %BF at 7 y (higher intake at 1 y) ▲BMIZ at 7 y (higher intake at 1 y) 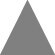 BMIZ at 7 y (higher intake between 1 and 2 y)	—	—	—	
**Garden et al** (2011)^31^	—	—	—	Carbohydrates intake at 18 mo: < > BMI at 8 y < > WC at 8 y	—	—	—	Low
**Huus et al** (2009)^33^	—	—	—	Candy intake at 2.5 y: < > Overweight/obesity at 5 y	—	—	—	Some
**Jardí et al** (2019)^34^	—	—	—	Free sugars at 12 mo: ▲WHZ at 30 mo < > WHZ at 12 mo Natural sugars, starches at 12 mo: < > WHZ at 12 mo	—	—	—	Some
**Generation R Study:**								
Kiefte de Jong et al (2013)^35^	—	—	Mono- and di- and polysaccharides at 14 mo: +/– Presence of constipation at 24, 36, 48 mo	—	—	—	—	Some
Nguyen et al (2020)^41^	—	—	—	Total carbohydrates; mono- and di- and polysaccharides at 12.9 mo: < > BMIZ at 10 y < > FMIZ, FFMIZ at 10 y	Total carbohydrates; mono- and di- and polysaccharides at 12.9 mo: < > Insulin at 10 y	Total carbohydrates at 12.9 mo: ▲TG < > TC, HDL-C, LDL-C at 10 y Mono- and disaccharides at 12.9 mo: ▲TG, HDL-C at 10 y < > TC, LDL-C at 10 y Polysaccharides at 12.9 mo: < > TC, HDL-C, LDL-C, TG at 10 y	—	Low
**Scaglioni et al** (2000)^43^	—	—	—	Carbohydrates intake at 1 y: < > Overweight at 5 y	—	—	—	Some
**Warner et al** (2006)^49^	—	—	—	Sweets consumption at 24 mo: < > Obesity at 2 y	—	—	—	Low
**Williams and Strobino** (2008)^50^	—	—	—	Dietary sucrose at 3-4 y: ▼BMI at 7-10 y	—	Dietary sucrose at 3-4 y: < > TC, HDL-C, TG at 7-10 y	—	Some
**SSBs and fruit juice**								
**Byrne et al** (2018)^25^	—	—	—	Mixed SSBs and fruit juice at 2, 3.7 y: < > BMIZ at 2, 3.7 y	—	—	—	Some
**Cantoral et al** (2016)^26^	—	—	—	SSBs only at 1-5 y: ▲Obesity at 8-14 y ▲Abdominal obesity at 8-14 y	—	—	—	Low
**Dubois et al** (2007)^30^	—	—	—	SSBs at 2.5, 3.5, and 4.5 y: ▲Obesity at 4.5 y	—	—	—	Low
**ECLS-B study:**								
DeBoer et al (2013)^29^	—	—	—	SSBs at 2 y: < > BMIZ at 2 y < > Overweight at 2 y < > Obesity at 2 y	—	—	—	Low
Shefferly et al (2016)^44^	Fruit juice at 2 y: ▲HAZ at 2-4 y 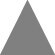 WAZ at 2-4 y	—	—	Fruit juice at 2 y: ▼BMIZ at 2 y, ▲ BMIZ at 2-4 y < > Overweight at 2 y, ▲Overweight at 2-4 y < > Obesity at 2 y, 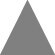 Obesity at 2-4 y	—	—	—	Low
Garden et al (2011)^31^	—	—	—	SSBs only and mixed SSBs and fruit juice at 18 mo: < > BMI at 8 y < > WC at 8 y	—	—	—	Low
**Generation R Study:**								
Leermakers et al (2015a)^37^	—	—	—	—	—	Mixed SSBs and fruit juice at 13 mo: 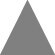 TG, HDL-C at 6 y < > TC, LDL-C at 6 y	—	Low
Leermakers et al (2015b)^36^	Mixed SSB and fruit juice at 13 mo: In boys at 6 y: ▼HAZ; < > WAZ In girls at 6 y: 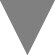 HAZ; 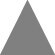 WAZ	—	—	Mixed SSBs and fruit juice at 13 mo: < > %BF at 6 y; 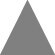 Higher android and gynoid ratio at 6 y in girls only ▲ BMIZ at 2, 3, 4, and 6 y in girls < > BMIZ at 6 y in boys	—	—	—	
**Lim et al** (2008)^38^	—	—	—	SSBs at 3-4 y: ▲ BMIZ at 3-4 y ▲ Overweight or obesity after 2 y of follow up	—	—	—	Low
**Quah et al** (2019)^42^	—	—	—	SSB at 18 mo: < > BMIZ, SSF at 6 y ▲, 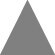 Overweight or obesity at 6 y	—	—	—	Some
**Viva Project study:**								
Sonneville et al (2015)^45^	—	—	—	Fruit juice at 1 y: < > WLZ at 1 y ▲ BMIZ at 3.1 y; 7.7 y	—	—	—	Low
Wu et al (2021)^51^	—	—	—	Fruit juice at 1 y: ▲ Abdominal adiposity at 8 y, 13 y	—	—	—	Low
**Warner et al** (2006)^49^	—	—	—	SSB at 2 y: ▲ Obesity at 2 y	—	—	—	Low
**WIC study:**								
Chaidez et al (2014)^27^	SSB at 12-24 mo: < > WAZ after 6 mo of follow-up	—	—	SSB at 12-24 mo: ▲ WHZ after 6 mo of follow-up ▲ BMIZ after 6 mo of follow-up	—	—	—	Some
Newby et al (2004)^40^	Fruit juice; SSBs at 2-5 y: < > Weight change after 6-12 mo	—	—	Fruit juice; SSBs at 2-5 y: < > BMI change after 6-12 mo	—	—	—	Some
**Nondigestible carbohydrates**								
**Dietary fiber**								
**ALSPAC study:**								
Cowin et al (2001)^28^	Fiber intake at 18 mo: < > Height at 31 mo	—	—	Fiber intake at 18 mo: < > BMI at 31 mo	—	Fiber intake at 18 mo: < > TC, HDL-C, LDL-C, TC:HDL-C at 31 mo	—	Low
Tappin et al (2020)^46^	—	—	Fiber intake at 2 y: ▼Stool frequency and consistency at 2.5 y ▼ Presence of constipation at 4-10 y	—	—	—	—	Low
Taylor et al (2016)^47^	—	—	Fiber intake at 38 mo: ▼Stool hardness (constipation) at 42 mo	—	—	—	—	Some
**Beyerlein et al** (2015)^23^	—	—	—	—	Fiber intake at 12 mo: < >T1DM; islet autoimmunity up to 5 y	—	—	Low
**Buyken et al** (2008) (DONALD)^24^	—	—	—	Fiber intake at 2 y: < > %BF, BMZ at 2 y; 2-7 y change	—	—	—	Some
**Jardí et al** (2019)^34^	Fiber at 12 mo: < > Excess weight at 12 mo < > Excess weight at 30 mo	—	—	—	—	—	—	Some
**Newby et al** (2003)^39^ (WIC)	Fiber intake at 2.9 y: < > Weight change after 6-12 mo	—	—	—	—	—	—	Some
**Generation R Study:**								
Kiefte de Jong et al (2013)^35^	—	—	Fiber intake at 14 mo: < > Presence of constipation at 24, 36, 48 mo	—	—	—	—	Some
Van Gijssel et al (2016)^48^	—	—	—	Fiber intake at 12.9 mo: < > %BF *z* score at 6 y	Fiber intake at 12.9 mo: < > Insulin *z* score at 6 y	Fiber intake at 12.9 mo: ▼HDL-C, TG at 6 y	—	Low
**Williams and Strobino** (2008)^50^	—	—	—	Fiber intake at 3-4 y: < > BMI at 7-10 y	—	Fiber intake at 3-4 y: ▼ TC < > TG, HDL-C at 7-10 y	—	Some
**Nakamura et al** (2006)^52^ (RCT) (2 g/d of fructo-oligosaccharides supplementation or placebo for 6 mo)	< > Weight gain, height, arm circumference during 6 mo	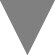 Number of diarrheal episodes during 6 m ▼ Duration of diarrheal episodes during 6 m	▼ Duration of diarrhea during 6 mo < > Stool frequency during 6 mo	—	—	—	—	Low
**Waligora-Dupriet et al** (2007)^53^ (RCT) (2 g/d of fructo-oligosaccharides supplementation or placebo for 21 d)	—	▼Diarrheal episodes during 28d ▼ Number of infectious diseases requiring antibiotic Treatment during 28d	< > Stool frequency, consistency, and abdominal pain during 28 d	—	—	—	▼Bifidobacteria and Clostridia levels at 28 d	Some

a Effect direction—beneficial (positive) effect: ▼ (black arrow; statistical significance, *P* < .05); 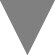 (gray arrow; statistical nonsignificance, *P* ≥ .05); negative (not-beneficial) effect: ▲ (black arrow; statistical significance, *P* < .05); 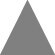 (gray arrow; statistical insignificance, *P* ≥ .05); no association/no change: < >; +/– conflicting results.

b Risk-of-bias assessment: based on the Mixed Methods Appraisal Tool (see Table S2A and S2B). “Low” risk of bias when ≤1 question was answered with “no” or “can’t tell”; overall risk of bias was considered as of “some concerns” when 2–3 questions were answered with “no” or “can’t tell”; overall risk of bias was considered “high” when 4–5 questions were answered with “no” or “can’t tell.”

Abbreviations: ALSPAC, Avon Longitudinal Study of Parents and Children; %BF, body fat percentage; BMI, body mass index; BMIZ, BMI *z* score; DONALD, Dortmund Nutritional and Anthropometric Longitudinally Designed Study; ECLS-B, Early Childhood Longitudinal Study, Birth Cohort; FFMIZ, fat-free mass index *z* score; FMIZ, fat mass index *z* score; HDL-C, high-density-lipoprotein cholesterol; LDL-C, low-density-lipoprotein cholesterol; RCT, randomized controlled trial; SSB, sugar-sweetened beverage; SSF, sum of skinfolds; T1DM, type 1 diabetes mellitus; TC, total cholesterol; TG, triglycerides; WAZ, weight-for-age *z* score; WC, waist circumference; WHZ, weight-for-height *z* score; WIC, Special Supplemental Nutrition Program for Women, Infants, and Children; WLZ, weight-for-length *z* score.

## RESULTS

**Figure 1.**
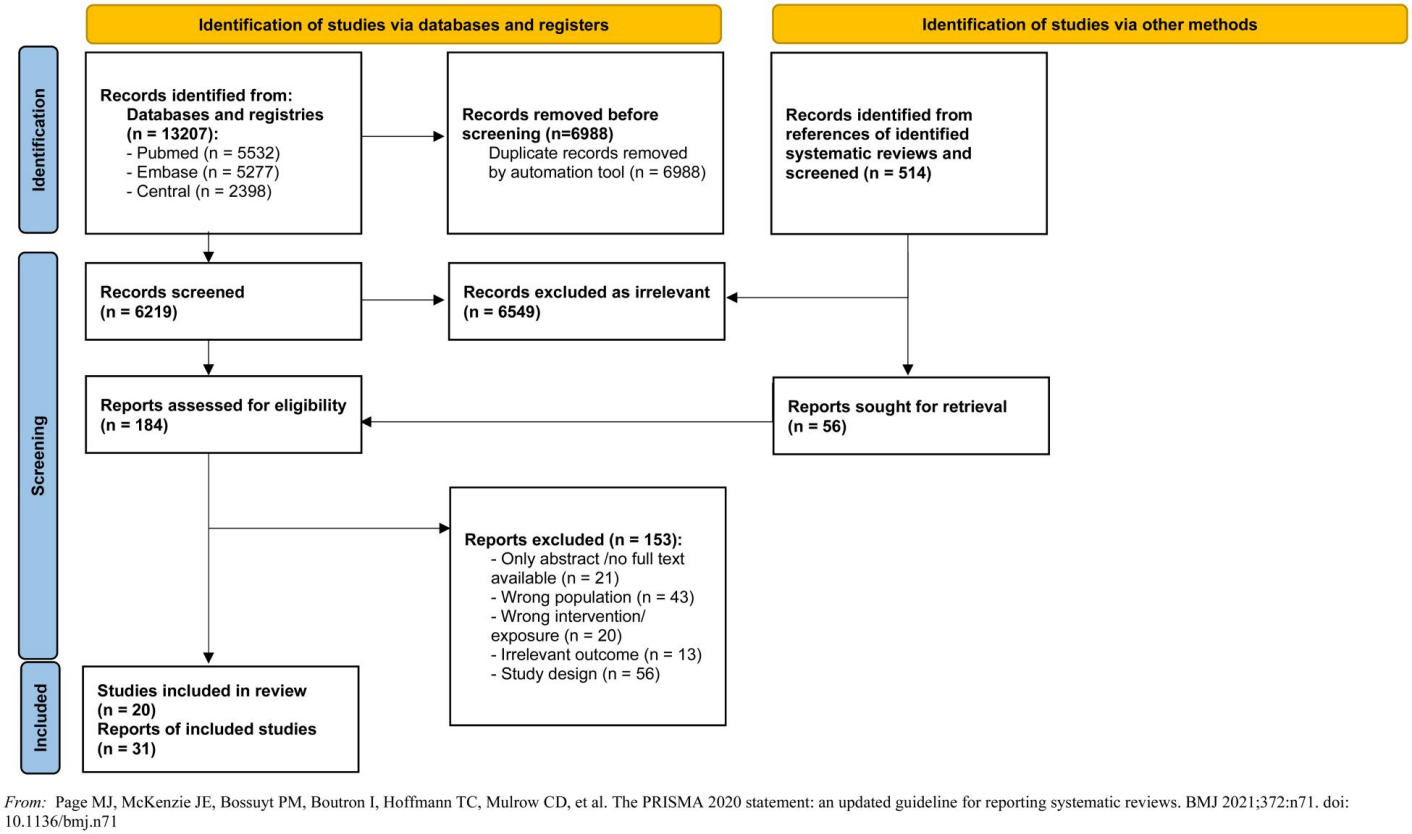
Flow Diagram of the Literature Search and Study Selection Process. Reproduced from reference 21

Figure 1 shows a flow diagram of the literature search and study selection process. A total of 13 207 records were obtained after applying the search strategy (**Table S1**). Of these, 184 full texts were screened, leading to the inclusion of 20 studies. The results of these studies were published in 31 papers,^23–53^ including 2 RCTs^52^^,^^53^ and 18 prospective cohort studies.^23–51^ Among the included studies, data from 6 prospective studies resulted in 17 publications included in this review: the Avon Longitudinal Study of Parents and Children (ALSPAC)^28^^,^^46^^,^^47^; the Early Childhood Longitudinal Study, Birth Cohort (ECLS-B)^29^^,^^44^; the Dortmund Nutritional and Anthropometric Longitudinally Designed Study (DONALD)^24^^,^^32^; the Generation R Study^35–37^^,^^41^^,^^48^; Project Viva^45^^,^^51^; and the Special Supplemental Nutrition Program for Women, Infants, and Children (WIC).^27^^,^^39^^,^^40^ Authors of these studies reported different exposures and outcomes in their publications. Therefore, we analyzed available data with a referral to the main study. Sample sizes of the included studies ranged from 20 to 13 954 at baseline, with 2 cohort studies of fewer than 200^34^^,^^43^ and 1 RCT with fewer than 40 participants.^53^ Various definitions of exposures and outcomes, whenever reported, were used in the original studies. The age of children at the time of outcome assessment varied from 1 year up to 14 years of age, and was within 2–6 years in most of the studies. The timing of outcomes assessment varied from immediate (in cross-sectional analyses) to short-term (up to 2–3 years of follow-up) and long-term (>3 years of follow-up) in longitudinal analyses. The number of children available for analysis differed based on outcomes of interest, as follows:

1. Growth: 4 prospective cohorts, *n* = ∼12 900 (ALSPAC,^28^ ECLS-B,^44^ Generation R,^36^ WIC^27^^,^^39^^,^^40^) and a single RCT,^52^  *n* = 150
2. Infections (2 RCTs),^52^^,^^53^  *n* = 170
3. Bowel function (ALSPAC,^46^^,^^47^  *n* = ∼9500; Generation R,^35^  *n* = 2420; 2 RCTs,^52^^,^^53^  *n* = 170)
4. Cognitive and psychomotor development: no studies identified
5. Obesity/overweight/body composition (assessed in almost all [17] studies, with the exception of 1 prospective cohort, The Environmental Determinants of Diabetes in the Young [TEDDY] study and 2 RCTs),^52^^,^^53^  *n* = ∼27 300
6. Glucose metabolism and homeostasis: 2 studies (Generation R,^41^^,^^48^ TEDDY study^23^), *n* = ∼4700
7. Lipids: 3 studies (ALSPAC,^28^ Generation R,^37^^,^^41^^,^^48^ Healthy Start Project^50^), *n* = ∼3200
8. Gut microbiota, *n* = 20 (RCT)^53^

References to the 153 excluded papers, with accompanying reasons for exclusion, are reported in **Table S3**.

### Risk-of-Bias Assessment

Details of MMAT assessment of risk of bias of observational studies (**Table S2A**) and of interventional studies (**Table S2B**) are presented in the supplementary materials. For the observational studies, only 1 study was considered as having a low risk of bias in all domains (TEDDY^23^). In others, a high risk of bias was present in at least 1 domain. In the sample representativeness, most of the included studies had a low risk of bias. Nevertheless, very small samples, convenience sampling method, or unrepresentativeness of the sample reported by the authors resulted in a high risk of bias for 5 studies, 8 publications.^24–27^^,^^32^^,^^34^^,^^39^^,^^40^ Exposure data were collected mostly through a parental questionnaire and, in some instances, by 24-hour dietary recall^25^^,^^27^^,^^34^^,^^50^ or by interview.^26^^,^^29^^,^^34^^,^^44^^,^^49^^,^^50^ The assessment of dietary intake varied in terms of (1) tools used by the authors of included studies (validation mostly not done/or reported) and (2) process of acquiring data (parental self-report vs interviews by trained assessors). Outcome data were measured by trained staff, with standardized protocols in most of the studies, with 4 exceptions of parental self-report.^33^^,^^35^^,^^46^^,^^47^ Incomplete outcome data were an issue in 5 studies (7 publications^27^^,^^31^^,^^32^^,^^38–40^^,^^50^). In most of the publications, adjustment for confounding factors in the analyses of our interest was done, with 3 exceptions.^25^^,^^35^^,^^47^ In 14 of the 31 included publications, potential confounding of infant feeding had been considered and breastfeeding duration was included as a covariate in statistical analyses.^23^^,^^24^^,^^26^^,^^31^^,^^32^^,^^35–37^^,^^41^^,^^42^^,^^46–49^ Breastfeeding duration was analyzed as an independent variable but not analyzed as potential confounders in 2 publications.^43^^,^^45^ Authors of 1 study estimated breast-milk intake via measuring the duration of breastfeedings in minutes, but did not include it as confounding factor in their analysis.^25^ In 2 publications, children who were still being breastfed at baseline were excluded.^40^^,^^52^ The intake of human milk was not addressed by authors of 12 publications.^27–30^^,^^33^^,^^34^^,^^38^^,^^39^^,^^44^^,^^50^^,^^51^^,^^53^ Among RCTs, 1 was of low risk of bias in almost all domains.^52^ In the second, appropriateness of randomization and provision of blinding were unclear, with a high risk of bias considered due to incompleteness of outcome data availability.^53^

### Digestible Carbohydrates

The effects of DC exposure were investigated in 17 studies, resulting in a total of 24 publications.^24–38^^,^^40–45^^,^^49–51^ Investigators of all studies addressed the outcomes of obesity, overweight, and body composition, and some of them addressed growth,^27^^,^^28^^,^^36^^,^^44^, bowel function,^35^ glucose metabolism and homeostasis,^41^ and blood lipids.^28^^,^^37^^,^^41^^,^^50^ The studies demonstrated generally “some” to “low” risk of bias. The authors used a variety of definitions of their studied exposures if defined at all. In this section, we have categorized exposures into 3 main groups: (1) total carbohydrates, mono- and disaccharides (including “sugars,” “diet sucrose,” “natural sugars,” as these terms typically align with mono and/or disaccharides), and polysaccharides (including “starch”); (2) added sugars, free sugars, and sweets consumption are grouped into the latter category, as they typically refer to specific sources or forms of sugars in the diet, often involving sugars added during food processing or the overall amount of sugars not naturally present in foods like fruits and vegetables; and (3) a subgroup of carbohydrates in liquid form in which large quantities are consumed very quickly (ie, SSBs and fruit juice intake).

### Total Carbohydrates, Mono- and Disaccharides, and Polysaccharides

#### Growth

In the ALSPAC study, a weak correlation was observed between increased energy-adjusted total carbohydrate (*r* = –0.14, *P* = .02) and higher energy-adjusted starch consumption (*r* = –0.12, *P* = .01), but not sugar (mono- and disaccharides), at 18 months and a subsequent decrease in height among boys, whereas no correlation was identified for girls’ height at 31 months.^28^

*In summary, a weak correlation between total carbohydrate or starch intake at 1.5 years and reduced height in the short-term (∼2.5 years) was observed, solely in boys.*

#### Bowel Function

In the Generation R Study (*n* = 2420),^35^ with some risk of bias, lower intake (in terms of energy percentage) of polysaccharides and higher intake of mono- and disaccharides at 14 months were associated with the presence of constipation among children aged 48 months (but not at 24 and 36 months).

*In summary, there is conflicting evidence on an association between mono-, di-, and polysaccharides intakes after the first year of life and constipation patterns from 2 to 4 years.*

#### Body Mass Index, Body Composition, Risk of Overweight and Obesity

In the prospective cohort from the Childhood Asthma Prevention Study (CAPS) comprising 362 children, the carbohydrate intake at 18 months was not a predictor for body mass index (BMI), nor did it influence waist circumference at 8 years, in adjusted analyses.^31^ Similarly, in the ALSPAC study (*n* not reported), no association was observed between elevated total carbohydrate, increased sugar, or starch intake at 18 months and BMI at 31 months.^28^ In a smaller prospective study (*n* = 81), the intake of natural sugars or starches at 12 months showed no association with excess weight, defined as weight-for-height *z* score (WHZ) greater than +1 *z* score. This lack of association was observed in both cross-sectional (at 12 months) and prospective (at 30 months) analyses.^34^ In the Generation R Study (*n* = ∼3100–3500, depending on the analysis), no associations between total carbohydrates, mono- and disaccharides, as well as polysaccharides with BMI *z* score (BMIZ), and body-composition measures (fat mass [FM] *z* score and fat-free mass [FFM] *z* score) at 10 years were noted.^41^ In a prospective cohort from Italy (*n* = 147),^43^ carbohydrate intake (in total) at 1 year was not associated with overweight (defined as >90th percentile of the Rolland-Cachera curve) at 5 years. Finally, in the Healthy Start Project study,^50^ which included 252 children, dietary sucrose (disaccharide) consumption at age 3–4 years was inversely associated with BMI at 7–10 years.

*In summary, no significant associations between total carbohydrates (including mono- and disaccharides, as well as polysaccharides) and immediate and later reported outcomes of BMIZ, body-composition measures (FM z score and FFM z score), and the prevalence of overweight were noted in most of the identified studies.*

#### Glucose Metabolism and Homeostasis

The Generation R Study was the only study to provide information on the intake of total carbohydrates and its various subcategories (monosaccharides, disaccharides, and polysaccharides) at 1 year, demonstrating no association with insulin levels at 10 years.^41^

*In summary, there was no observed association between total carbohydrates intake at 1 year (including its subcategories) and insulin levels at 10 years.*

#### Blood Lipids

In the Generation R Study,^41^ the intake of total carbohydrates and mono- and disaccharides at 1 year was associated with elevated triglyceride (TG) concentrations at 10 years. The intake of mono- and disaccharides alone was linked to lower high-density-lipoprotein cholesterol (HDL-C) levels. However, no associations were found between total and mono- and disaccharides intake at 1 year and subsequent total cholesterol (TC) or low-density-lipoprotein cholesterol (LDL-C) levels. Polysaccharide intake at 1 year was not associated with any subtype of lipids at a later age. In the univariate analyses of the ALSPAC study,^28^ some weak correlations (*r* < 0.3), exclusively observed in boys, indicated associations with total carbohydrate intake at 18 months (with lower TC and a higher TC:HDL ratio at 31 months) and sugar intake (mono- and disaccharides associated with higher TC levels at 31 months). However, no associations were identified between starch (polysaccharide) intake and lipid components at 31 months. Subsequently, in the multivariate analysis, a negative association between sugar intake at 18 months and HDL-C at 31 months was observed specifically in girls (*P* = .05), with no other correlations identified for total carbohydrate, sugar, and starch intake in this group. Additionally, in the Healthy Start Project study,^50^ which included 252 children, dietary sucrose (disaccharide) consumption at the age of 3–4 years was not associated with any of the lipid components at 7–10 years.

*In summary, the largest study, with a low risk of bias, indicated some associations between elevated early carbohydrate intake and subsequent higher TG levels, as well as lower HDL-C, while 2 smaller studies showed limited or no such associations.*

### Added Sugars, Free Sugars, Sweets

#### Growth

The intake of added sugars at 12–24 months in a limited sample (*n* = 67) from the WIC study showed no association with subsequent changes in weight-for-age *z* score (WAZ) after 6 months.^27^

*In summary, there were no immediate weight changes associated with added-sugars intake, as indicated by a single small study.*

#### BMI, Body Composition and Risk of Overweight and Obesity

The intake of added sugars at 12–24 months in a limited sample (*n* = 67) from the WIC study showed no association with subsequent changes in WHZ and BMIZ after 6 months.^27^ In the DONALD study (*n* = 216–380),^24^^,^^32^ added-sugar intake was evaluated as an exposure at 3 time points: at 1 year, the change within 1–2 years, and 2 years. The findings indicated that higher added-sugar intake at 1 year was associated with lower BMIZ but not percentage of body fat (%BF) at 7 years. Changes in intake during the second year showed a trend towards higher BMIZ, but no association with %BF at 7 years. Moreover, added-sugar intake at 2 years exhibited no association with %BF and BMIZ both immediately (cross-sectional analysis) and longitudinally, considering changes in these parameters between 2 and 7 years. Free-sugars intake at 12 months in 81 children showed a prospective association with excess weight (WHZ > +1 *z* score) at 30 months, but not in the immediate, cross-sectional analysis at 12 months.^34^ Two studies were found that examined the relationship between sweets consumption and overweight/obesity status. In the first study involving 7356 children, consumption of less than 1 time per week vs 3–5 times per week of candies at 2.5 years was not associated with overweight/obesity at 5 years.^33^ In the second study, daily consumption of 1 or more sweets (including candy, cookies, ice creams, or pan dulce) at 24 months did not increase the odds of overweight at the same time, when compared with consuming less than 1 sweet per day in 354 analyzed children.^49^

*In summary, among the included studies that assessed added-sugar, free-sugars, and sweets intake early in life, no significant immediate short-term or later effects on growth, overweight, and obesity indices were noted.*

### Liquid Carbohydrates

Authors of 11 studies in 15 publications^25–27^^,^^29–31^^,^^36–38^^,^^40^^,^^42^^,^^44^^,^^45^^,^^49^^,^^51^ assessed SSBs and/or fruit juice intake as an exposure of interest. It is important to note that studies focusing solely on liquid carbohydrates may be limited in scope, as they represent a specific type of DC that may not fully reflect total carbohydrate or sugar intake. Additionally, the liquid form of SSBs or fruit juice introduces potential confounding factors, such as rapid absorption rates and differing metabolic responses compared with solid food sources. These limitations should be considered when interpreting the findings of studies involving liquid carbohydrates.

### Sugar-Sweetened Beverages

The impact of SSBs as the only exposure was investigated in 8 prospective cohort studies, comprising a total of 9 publications.^26^^,^^27^^,^^29–31^^,^^38^^,^^40^^,^^42^^,^^49^ The studies primarily focused on assessing the effects of SSB intake on obesity, overweight, and body composition, with only 1 of them (in 2 publications) addressing growth outcome.^27^^,^^40^

#### Growth

The authors of the WIC study found that annual weight change in 1345 children remained unaffected by the type of SSB intake,^40^ and in a small sample of 67 children aged 12–24 months, higher SSB intake did not impact the WAZ after a 6-month period.^27^

*In summary, no association between SSB consumption and child growth was observed, based on the results of a single study with some concerns regarding the risk of bias.*

#### BMI, Body Composition and Risk of Overweight and Obesity

A total of 8 studies examined the impact of SSB intake (9 publications)^26^^,^^27^^,^^29–31^^,^^38^^,^^40^^,^^42^^,^^49^ with the exclusion of fruit juice from the definition. Of these, 6 studies had a low risk of bias, based on the MMAT assessment.^26^^,^^29–31^^,^^38^^,^^49^ In 3 studies (CAPS, *n* = 362; ECLS-B, *n* = 7600; Growing Up in Singapore Towards healthy Outcomes (GUSTO) cohort study, *n* = 555),^29^^,^^31^^,^^42^ no significant effects on BMI and BMIZ were observed. These studies assessed SSB exposure around the second year of life, with outcomes evaluated immediately at 2 years, and prospectively at the ages of 6 and 8 years. In the largest study involving 7600 children at the time of outcome assessment,^29^ a cross-sectional analysis did not reveal any immediate effects of SSB intake on evaluated BMIZ, overweight, or obesity at the age of 2 years. However, the GUSTO study (*n* = 555) presented different results based on the analytical methods used. In 1 analysis, when SSB intake at 18 months was modeled in 100-mL/d increments, an increased risk of overweight/obesity at 6 years was observed. This effect was not seen in relation to BMIZ or the sum of skinfolds (SSF). In a separate analysis within the same study, when comparing groups that were defined by varying levels of SSB intake, no statistically significant associations were identified with BMIZ, SSF, or the risk of overweight/obesity. Within the WIC study,^27^^,^^40^ no significant annual change in BMI was observed among 1345 children. Conversely, in a separate publication from the same study, an increase in BMIZ and WHZ was reported after a 6-month follow-up period in a small sample of children. Nonetheless, a subset of the studies included in this section, with a relatively smaller combined sample size of approximately *n* = 2400 at the time of outcome assessment,^26^^,^^30^^,^^38^^,^^49^ consistently reported negative (unbeneficial) associations with SSB intake. In these specific studies, the consumption of SSBs during early childhood (1–4 years) was associated with subsequent outcomes such as abdominal obesity (at 8–14 years),^26^ greater BMI (∼5 years),^38^ obesity (at 2–14 years),^26^^,^^30^^,^^49^ and overweight (∼2 years of follow-up).^38^ These discrepancies in results could not be explained by the methodological differences.

*In summary, no significant associations were found between SSB intake and immediate outcomes of interest. However, early SSB consumption during the ages of 1–4 years was associated with unfavorable short- and long-term effects on BMI, body composition, and obesity in a subset of included studies, although discrepancies were present in some other studies.*

### Fruit Juice

The impact of fruit juice exposure was investigated in 3 studies, comprising a total of 4 publications, with “low” to “some” risk of bias.^40^^,^^44^^,^^45^^,^^51^ All studies focused on assessing the effects of fruit juice intake on obesity, overweight, and body composition, with 2 studies addressing growth outcome.^40^^,^^44^

#### Growth

In the ECLS-B study, which comprised 8950 children, those who regularly consumed fruit juice at the age of 2 years (≥1 serving/d) experienced a decrease in height-for-age *z* score (HAZ) between 2 and 4 years, accompanied by a trend towards higher WAZ, that did not reach statistical significance (*P* = .06).^44^ Additionally, no association with annual weight change was observed in the 1379 children who consumed fruit juice in the WIC study.^40^

*In summary, the findings from a single study with a low risk of bias revealed a decrease in HAZ in children between the ages of 2 and 4 years. No significant association between fruit juice consumption and WAZ was reported in both included studies.*

#### BMI, Body Composition, and Risk of Overweight and Obesity

Three prospective cohort studies, in 4 publications,^40^^,^^44^^,^^45^^,^^51^ evaluated the influence of fruit juice consumption on BMI and the risk of overweight and obesity. In the ECLS-B study, which involved 8950 children, a cross-sectional analysis at the age of 2 years did not reveal immediate differences in the risk of overweight/obesity but showed an association with lower BMIZ among children who regularly consumed fruit juice. However, in a prospective analysis, regular fruit juice consumption at 2 years was associated with increased BMIZ and an elevated odds ratio of overweight between the ages of 2 and 4 years, although it did not significantly influence the risk of obesity as compared with infrequent/non-drinkers.^44^ In the Viva project, with a sample size of 783, no immediate associations were observed in a cross-sectional analysis of fruit juice intake at 1 year and weight-for-length *z* score (WLZ). However, higher fruit juice intake at 1 year was associated with increased BMIZ during early and mid-childhood (at 3 and 7 years, respectively), as well as consistently greater visceral, subcutaneous, and total abdominal adipose tissue, measured at 8 and 13 years in prospective analyses.^45^^,^^51^ The 1379 children participating in the WIC study did not demonstrate any association between fruit juice consumption and annual BMI changes.^40^

*In summary, no significant associations were found between fruit juice consumption and immediate outcomes of interest. Nevertheless, early fruit juice intake around the age of 1 year was linked to unfavorable long-term effects on BMI, body composition, and overweight (excluding obesity).*

### SSBs (Including Fruit Juice in the Definition)

The effects of combined exposure of fruit juice and SSBs were investigated in 3 studies, resulting in a total of 4 publications.^25^^,^^31^^,^^36^^,^^37^ The studies demonstrated a generally low risk of bias, although 1 study had “some” risk of bias.^25^ Investigators addressed the outcomes of growth,^36^ body composition, BMI,^25^^,^^31^^,^^36^ and lipid profiles.^37^

#### Growth

In the Generation R Study, which involved 2371 children, it was observed that, in boys, a higher intake of sugar-containing beverages (SCBs) at 13 months was associated with increased height at the age of 6 years, with no significant impact on WAZ. Conversely, in girls, there was a tendency for SCB intake to be associated with both WAZ and HAZ at 6 years.^37^

*In summary, based on the results from the single low-risk-of-bias study, there was no consistent pattern of association between increased consumption of SCBs at 13 months and growth outcomes in the long term.*

#### BMI, Body Composition, and Risk of Overweight and Obesity

In a cohort of 362 children from Australia, the association between non-milk beverage intake at 18 months of age and waist circumference at 8 years did not maintain statistical significance after adjustment.^31^ Similarly, in the Generation R Study involving 2001 children, no significant associations were found between SCB intake at 13 months and body fat percentage at 6 years, although there was a trend towards a higher android:gynoid fat ratio in girls.^36^ In terms of BMI outcome, most of the analyses reported no differences. Longitudinal analyses of BMIZ changes at 2 and 3.7 years in 426 children showed no significant relationship with SSB, including fruit juice, intake.^25^ Additionally, no differences were observed in prospective analysis of a second study that examined exposure of non-milk beverage consumption at 18 months and BMI at 8 years.^31^ In the Generation R Study, in a subset of girls only, a higher SCB intake at 13 months was significantly associated with consistently higher BMI at ages 2, 3, 4, and 6 years (at age 6 years, BMIZ increase of 0.11).^36^

*In summary, the majority of included studies did not show a significant association between the combined group of mixed SSBs and fruit juice and subsequent BMI or body composition outcomes. Nonetheless, in a specific subset of girls from a single study with low risk of bias, a consistent and notable finding*  *of unfavorable effects associated with mixed SSB and fruit juice exposure was observed. No studies assessed overweight or obesity as specific outcomes of interest.*

#### Blood Lipids

An analysis of 1390 children from the Generation R Study, with low risk of bias, indicated a trend towards elevated concentrations of TG and reduced HDL-C levels at the age of 6 years. This trend was observed among children in the highest tertile of SCB intake at 18 months, with no significant change in LDL-C and TC levels.^37^

*In summary, a trend was observed towards increased TG and decreased levels of HDL-C at the age of 6 years in a single, low-risk-of-bias study. This trend was seen among children with the highest tertile of combination of both SSB and fruit juice consumption at 13 months, compared with the group with the lowest intake. No associations were noted in relation to TC and LDL-C.*

### Nondigestible Carbohydrates (Dietary Fiber)

The associations of NDC intake (dietary fiber) effects were investigated in 7 prospective cohort studies (10 publications)^23^^,^^24^^,^^28^^,^^34^^,^^35^^,^^39^^,^^46–48^^,^^50^ and in 2 RCTs, in which the FOS supplementation effects were compared with placebo.^52^^,^^53^

Among these publications, 3 reported data from the ALSPAC study.^28^^,^^46^^,^^47^ The studies examined a wide range of our outcomes of interest, including the following: growth,^28^^,^^34^^,^^39^ bowel function,^35^^,^^46^^,^^47^ BMI or body composition,^24^^,^^28^^,^^48^^,^^50^ glucose homeostasis and metabolism,^23^^,^^48^ and lipid profiles.^28^^,^^48^^,^^50^ Among these publications, 4 were assessed as having “low” risk of bias,^23^^,^^28^^,^^46^^,^^48^ while others had “some” risk of bias. Additionally, 2 RCTs with “low” to “some” risk of bias assessing the effects of FOS on growth, infection, bowel function, and microbiota were reported separately.^52^^,^^53^

#### Growth

In the WIC study, there was no observed association between total daily fiber intake and the annual weight change in 1379 children, followed up after 6–12 months from baseline.^39^ Similarly, in a small Spanish study involving 81 children, higher fiber intake at 12 months was not linked to increased probability of excess weight. This was observed both immediately and prospectively assessed up to 30 months.^34^ Furthermore, in the ALSPAC study, fiber consumption at 18 months was not found to be associated with height at 31 months.^28^

*In summary, there were no observed associations between dietary fiber intake and all reported growth outcomes.*

#### Bowel Function

The ALSPAC study^46^^,^^47^ provided insights into bowel function and constipation-related outcomes. Tertiles of fiber intake at 38 months were significantly associated with parentally reported stool hardness in the short term at 42 months. Children with the lowest fiber intake were nearly twice as likely to have “usually” hard stools compared with those with the highest fiber intake.^47^ Furthermore, the study revealed a significant association with hard stools at 2.5 years and longitudinal patterns of constipation at ages 4–10 years, specifically with lower fiber intake at 2 years. However, the authors suggested that the early signs of constipation, such as hard stools, likely precede the weaning period, and therefore, the observed association between low fiber intake and constipation may not be directly causal.^46^ In the Generation R Study, dietary fiber intake at 14 months was not associated with parentally reported constipation in children at 24, 36, and 48 months.^35^

*In summary, reduced dietary fiber intake at the age of 1–2 years was associated with short-term endpoints, such as the presence of hard stools; however, associations with constipation patterns extending from ages 2 to 10 years were not consistently reported. Moreover, reverse causality cannot be excluded.*

#### BMI, Body Composition, and Risk of Overweight and Obesity

In the Generation R Study, which involved 1380 children and assessed fiber intake at 12.9 months, no association was found with the %BF *z* score measured by dual-energy X-ray absorptiometry (DXA) at 6 years.^48^ Similarly, in the ALSPAC study (*n* = 389), fiber consumption at 18 months was not associated with BMI at 31 months.^28^ Likewise, in the DONALD study (*n* = 380) fiber intake at 2 years was not linked to BMIZ both immediately in cross-sectional analysis or with BMIZ changes between 2 and 7 years, and there were no significant associations with changes in %BF between 2 and 7 years, as measured by skinfold thickness differences.^24^ Additionally, in the Healthy Start Project study, which included 252 children, fiber consumption at the later age of 3–4 years was not associated with BMI between ages 7 and 10 years.^50^

*In summary, based on 4 PCs with some to low risk of bias, no associations were consistently observed between dietary fiber intake in children aged 1–4 years and both immediate and later outcomes related to BMI or body composition. No studies assessed overweight or obesity as specific outcomes of interest.*

#### Glucose Metabolism and Homeostasis

In the TEDDY study, which included approximately 3300 children with analyzed dietary fiber consumption at 12 months, there was no observed association with the development of islet autoimmunity or type 1 diabetes mellitus (T1DM) during the 5-year follow-up period.^23^ Similarly, in the Generation R Study, dietary fiber intake at 12.9 months was not linked to insulin *z* score at 6 years among 1380 children.^48^

*In summary, 2 PCs with low risk of bias, investigating particular outcomes, did not identify any associations between dietary fiber intake at approximately 1 year of age and the development of T1DM, islet autoimmunity, or with insulin z score during the 5–6 years of follow-up.*

#### Blood Lipids

In the ALSPAC study, dietary fiber intake was assessed in a randomly selected group of children (214 boys and 175 girls), and no association was found with TC, LDL-C, HDL-C, or the TC:HDL-C ratio at 31 months.^28^ In the Generation R Study, higher fiber intake among 1380 children at 12.9 months was associated with increased levels of HDL-C and lower TG *z* score concentrations at 6 years.^48^ A beneficial effect was also noted in the Healthy Start Project study, which included 252 children, in which dietary fiber consumption at the age of 3–4 years was associated with decreased TC levels between ages 7–10 years, although it had no effect on HDL-C or TG levels.^50^

*In summary, a generally beneficial trend of higher dietary fiber intake in children at ages of approximately 1–4 years and its association with later lipid levels among the included studies (which exhibited “low” to “some” risk of bias) was reported.*

### Nondigestible Carbohydrates (Dietary Fiber—RCTs)

The only 2 RCTs identified assessing effects of FOS, both exhibiting “low” to “some” risk of bias, involved different populations. The first trial included 150 children aged 25–59 months from an urban slum community in Bangladesh,^52^ and assessed impact on body-weight gain and reduction in diarrheal infections as primary outcomes. The second involved 20 children attending daycare centers in France with a median age of 12–14 months that studied impact on stool microbiota, gastrointestinal tolerance symptoms, and well-being (eg, use of medication).^53^ These trials assessed the effects of a daily dose of 2 g of FOS or oligofructose supplementation for varying durations of 6 months or 21 days and compared the effects with a placebo control.

#### Growth

In the cohort of urban slum children receiving FOS or placebo, 1 of the primary outcomes of interest was the improvement in body-weight gain over a 6-month supplementation period, and this did not exhibit a statistically significant difference (no *P* value reported).^52^ Measurements of height or arm circumference also did not change. There was also no change in growth reported for the healthy daycare children.^53^

*In summary, based on 1 RCT with low risk of bias, no substantial variations (ie, improvement in growth indices) were observed between the FOS supplementation and placebo groups after a 6-month supplementation period.*

#### Infection

The RCT with the urban slum children reported lack of a statistically significant effect on the number of diarrheal episodes (mean: 1.3 vs 2.0 in FOS and placebo groups, respectively, and a trend for a difference, *P* = .10). However, there was a significant reduction in the cumulative duration of these episodes (3.3 days in the FOS group vs 6.3 days in the placebo group; *P* = .01) over the 6-month supplementation period. In the 20 healthy daycare children, although not the primary objective of the study, the authors did observe a statistically significant decrease in the occurrence of infectious diseases requiring antibiotic treatment, as well as a reduced incidence of reported diarrheal episodes during the 21 days of the supplementation period within the oligofructose-supplemented group.^53^

*In summary, based on 2 RCTs with low to some risk of bias, a statistically significant difference was observed in reducing the number of infections in the short term, but not over an extended duration between the FOS-supplemented and placebo groups*.

#### Bowel Function

In both RCTs, with urban slum children or healthy children in daycare, an evaluation of bowel function was done. A significant reduction in the mean duration of diarrheal episodes by more than half a day was reported for the urban slum children in the FOS group.^52^ This reduction could be potentially significant for individuals. In the healthy daycare children, the number of episodes of diarrhea, flatulence, and vomiting were significantly lower in the oligofructose group compared with the control group. No statistically significant differences were observed in terms of stool frequency and consistency.^53^

*In summary, based on results from a single RCT characterized by a low risk of bias, a potentially clinically significant reduction in the duration of diarrhea was noted in the FOS-supplemented group.*

#### Gut Microbiota

The single study investigating this outcome in healthy children in daycare revealed that, during the 21-day supplementation period with 2 g per day, there was an observable trend (*P* ≤ .10) in the oligofructose group towards an increase in bifidobacteria levels and a decrease in Clostridia levels when compared with the placebo group based on bacteriological analysis.^53^

*In summary, differences in microbiota patterns did not reach statistically significant levels observed after a 21-day supplementation of FOS compared with a placebo among children in a daycare setting.*

## DISCUSSION

### Summary of Evidence

In this review we aimed to systematically evaluate the impact of both DC and NDC intake during early childhood (1–4 years of age) on various health outcomes. We identified significant associations in certain interventions/exposures, while many other dietary exposures did not appear to have a substantial impact on immediate or long-term health outcomes.

The intake of DCs, encompassing total carbohydrates, mono- and disaccharides, and polysaccharides, was not significantly associated with short-term or later outcomes related to growth, body composition, and the risk of overweight, indicating that these macronutrients, in isolation, may not have a pronounced direct impact on child health outcomes as previously speculated. However, the review indicated some associations between elevated early carbohydrate intake and subsequent higher TG levels, as well as lower HDL-C, in the largest study with a low risk of bias. Notably, early exposure to DCs in their liquid form of SSBs and fruit juice between the ages of 1 and 4 years was linked to unfavorable long-term effects on BMI, body composition, and the risk of obesity. Specifically, the early consumption of SSBs was associated with negative long-term health impacts, although these findings were not consistently observed across all studies. Similarly, early fruit juice intake was related to adverse long-term outcomes for body composition and an increased risk of overweight, albeit not affecting obesity rates directly.

Conversely, our review found no significant immediate adverse effects of SSB and fruit juice consumption on health outcomes, such as growth and immediate BMI changes, suggesting that the detrimental impacts of these beverages may manifest more significantly over time.

Nondigestible carbohydrates, as dietary fiber or soluble fiber intake from various food sources and FOS supplementation, showed no observed associations with growth indices, gastrointestinal tolerance symptoms, BMI, body composition, the development of T1DM, islet autoimmunity, or alterations in insulin *z* scores, highlighting a potential area for further investigation due to their importance in digestive health and metabolism. Supplementation with FOS/oligofructose in RCTs was associated with a reduction in the duration of diarrheal days and episodes and with fewer infections.

### Strength and Limitations

The strengths of this review process are in the use of a standardized procedure and the broad inclusion criteria, with prioritized outcomes selected by multiple experts from a cross-disciplinary team. The wide range of included studies resulted in a post hoc exposure assessment, which, while practical, may have introduced subjectivity in the grouping of studies. Defining subgroups after study inclusion could increase heterogeneity within the newly formed subcategories and inadvertently increase the risk of confirmatory bias.^54^ Furthermore, certain dietary assessment methods used in the included studies may not adequately distinguish between exposures of interest (eg, natural vs added sugars), potentially leading to misclassification within subgroups. We hypothesized that the identification of only a small number of studies would limit the ability to adequately address our research objectives. Thus, this idea of post hoc grouping was implemented to enhance the relevance of the findings for public health practice and policy, as the subcategories we established (eg, liquid carbohydrates, dietary fiber) reflect practical considerations. Moreover, a liquid carbohydrate subgroup was distinguished as it promotes positive energy balance due to incomplete compensation of energy intake when compared with sugars in solid form.^55^ We did not identify any studies conducted in middle- or low-income countries, where food behaviors have undergone significant nutritional transitions in recent years, including a dramatic increase in SSB consumption beginning at early ages.^56^

In this review, the evaluation of causality is limited, since almost all included studies are observational prospective cohorts. In addition, in some cases, the data were extrapolated from a main study not specifically designed to investigate the effects of the consumption of specific foods (eg, SSBs or fruit juice) on health outcomes.

Moreover, in both DC and NDC studies, the methods for dietary assessment varied widely across studies (food-frequency questionnaire, 24-hour recall, and 3-day diary; some of which were not standardized). It should be noted that different methods to measure dietary fiber, DCs, including their liquid form and especially in children’s diets, may have some effects on the results of the prospective cohort studies described. The included studies assessed a range of health outcomes, from constipation to blood lipid levels. While most analyses were longitudinal, there were differences in the participants’ age at follow-up, and some analyses were cross-sectional. With regard to DCs, the terminology used to refer to “added sugar” is often not further defined, with some studies limiting their assessment to specific food items like candy and sweets. Last, the portion size of liquid carbohydrates was not always defined, with intake based on weekly or daily frequency rather than precise quantities. These methodological discrepancies prevented the possibility of conducting a meta-analysis. While the risk of bias was low in most studies, concerns arose regarding sample representativeness, incomplete outcome data, and inconsistent adjustments for confounding factors, particularly the duration of breastfeeding. Among the RCTs, 1 study exhibited low risk of bias, whereas the other raised concerns regarding randomization procedures and outcome reporting. These limitations should be carefully considered when interpreting the findings of this review.

### Agreement and Disagreement With Other Studies or Reviews

#### Digestible Carbohydrates

Reviews of DC intake beyond SSBs in toddlers aged 1 to 4 years and related health outcomes are scarce. The WHO commissioned a review^57^ on the influence of increased dietary sugar intake on childhood overweight and obesity. The authors reported positive and negative associations for 15 and 4, respectively, out of 23 included cohort studies. Most of these 23 cohort studies related to SSB intake and included children and adolescents. Only 2 of them^29^^,^^44^ evaluated sugar intake in children between 1 and 4 years old and both reported a negative association. Another, more recent WHO-commissioned systematic review^58^ assessed the consumption of unhealthy foods, including 2 studies on added sugar or sweetened foods in children from 2 to less than 5 years and found insufficient evidence of an increase in BMI, %BF, or risk of overweight/obesity, concurring with the findings of the present study for children aged 1 to 4 years. A previous systematic review found positive associations between SSB consumption and BMI.^59^ However, the review included both longitudinal and cross-sectional studies and therefore no causal associations can be drawn. Another systematic review that estimated the effect of SSB intake in children and adolescents reported an increase in BMI by 0.07 (95% CI: 0.01, 0.12) for each additional daily serving of approximately 300 mL of SSBs using a random-effects model. However, it has to be considered that, in this systematic review, the reported heterogeneity was high (*I^2^* = 91.6%, *P* < .001).^60^ Recently, WHO performed a meta-analysis^58^ on SSB consumption and effects on BMI, body composition, and risk of overweight and obesity among children under 10 years of age and found a positive association between SSB consumption and body fat and BMIZ. However, the results should be interpreted with caution because the systematic review included a subsample of studies reporting a high bias risk. Last, a systematic review and meta-analysis on 100% fruit juice^61^ consumption in childhood and adolescence and BMI change concluded that, in children ages 1 to 6 years, a 1-serving (170 to 220 mL) increment was associated with a 4% increase in BMI percentile (95% CI: 0.008 to 0.167) and not associated with BMIZ increase in children ages 7 to 18 years.

#### Nondigestible Carbohydrates (Dietary Fiber)

We highlight findings from other reviews on NDCs; however, due to age differences, these findings remain indirectly related to the population assessed in our review. One systematic review and meta-analysis focused on the potential role of prebiotics in the prevention of acute infectious diseases in children aged 0–24 months.^62^ Based on the pooled analysis of 3 studies, a significant reduction in the number of infectious episodes requiring antibiotic therapy was observed in the prebiotic vs the placebo group. In 2017, experts reviewed the effects of fiber and prebiotics on functional gastrointestinal disorders and the microbiome in children.^63^ One of the main conclusions, that nutritional strategies with fiber or prebiotics may support gastrointestinal health in children,^63^ aligns well with the observations made in the present review.

Recent data, such as those presented by Basuray et al,^64^ demonstrate that increasing dietary fiber intake in pediatric populations can lead to improved metabolic outcomes, including better glycemic control and enhanced satiety. Although these studies involve slightly different populations, the findings offer valuable insights into how dietary fiber can play a crucial role in mitigating risk factors for pediatric obesity, such as excess adiposity. These results further emphasize the importance of addressing the “fiber gap” in children to improve long-term health outcomes.

### Implications for Practice/Research

To date, SSB consumption has been examined in children or adolescents only by comparing groups (consumers vs non-consumers). Randomized controlled trials or long-term studies following toddlers into adolescence and adulthood are needed to test whether unhealthy patterns of liquid carbohydrate intake in toddlers could have a long-term unfavorable impact on health outcomes, dietary patterns, and taste preferences.

There are also no RCTs so far studying the impact of total or specific dietary fibers on parameters such as overweight, lipid metabolism, and improved bowel habit/constipation issues in young children. Dietary fiber might be expected to have some effect on bowel habit in children, notably preventing or improving constipation issues,^65^ as this is one of the reasons dietary fiber is recommended at a certain level for adults.^66^ Nondigestible oligosaccharides appear to support a stool softening effect in infants.^67^ Two of the 3 cohort studies suggest there might be some association of dietary fiber with softer stool consistency, although it was suggested that the hard stools may precede and predict later fiber intake.^49^^,^^50^ It could be interesting to study the fiber/NDC levels and the intestinal microbiota from infants to young children to investigate this latter hypothesis. Further intervention studies, including mechanistic aspects such as analysis of the gut microbiota and their genome and metabolites, may support our understanding of the impact of total and different types of dietary fiber on bowel habits and infections in young children.

The current literature provides little information on health effects of total DCs or consumption of mono- and disaccharides from food during toddlerhood. Added-sugar intake in children is mostly studied in the form of SSBs. To create a more robust evidence base in regard to DCs in their solid form, high-quality epidemiological studies are needed that are designed to assess their effects during toddlerhood on disease risks and risk of overweight/obesity. As it is speculated that SSBs induce excessive energy intake by not promoting satiety compared with the equivalent amount of sugar in solid form,^5^ it could also be of interest to compare the energy compensation of young children after the consumption of sugar in its solid and liquid form.

Overall, there is a lack of studies, particularly RCTs, to comprehensively assess the effect of DC and NDC intake in toddlers on infections, bowel function, gut microbiota, as well as on cognitive and psychomotor development, glucose homeostasis, lipid metabolism, and other crucial health outcomes, both in the short and the long term.

## CONCLUSION

The negative impact on body weight and the risk of being overweight is less evident for total DCs and sugars in solid form compared with sugar intake in liquid forms, such as in SSBs and/or fruit juice. This finding is based on a relatively small number of studies.

The important finding emerging from our results is that the consumption of SSBs and fruit juice between the age of 1 and 4 years could have a negative impact on health outcomes later in life. These findings collectively emphasize the importance of monitoring and reducing SSB intake in toddlers to potentially mitigate the risk of altered body composition and potential associated health outcomes later in life. Therefore, dietary advice should be offered to parents that stresses the significance of reducing frequent exposure to these types of drinks in early life.

It is noteworthy that a general beneficial trend of higher NDC/dietary fiber intake in children at ages 1–4 years on later lipid levels was reported. Further studies are required to comprehensively assess the effect of DC and NDC intake in toddlers on cognitive and psychomotor development, infections, bowel function, and gut microbiota.

## Supplementary Material

nuae212_Supplementary_Data
