# Supplementary material for: ILSI Europe Systematic Review: The Impact of Digestible and Nondigestible Carbohydrate Consumption for Toddlers (1–4 Years) in Relation to Health Outcomes
Source: Nutr Rev. 2025 Feb 5;83(6):1099–132. doi: 10.1093/nutrit/nuae212 (PMC12066953; doi:10.1093/nutrit/nuae212)
Supplement: nuae212_Supplementary_Data [file nuae212_supplementary_data.zip › nuae212_Supplementary_Data/Supporting information_1203.docx]

**Supporting information:**

Table S1. Search strategy (MEDLINE via Pubmed)

Table S2a. MMAT assessment of risk of bias of observational studies

Table S2b. MMAT assessment of risk of bias of Randomized Controlled Trials

Table S3. Excluded studies table

**Table S1. Search strategy (MEDLINE via Pubmed)**

| ((((((((("Obesity"[Mesh]) OR "Overweight"[Mesh]) OR "Body Size"[Mesh]) OR "Body Constitution"[Mesh]) OR "Body Weights and Measures"[Mesh]) OR (((((((((((((((((((((((((((((((obesity) OR (obese)) OR (overweight)) OR (weight)) OR (weights)) OR ("body mass index"[All Fields])) OR (bmi)) OR (growth)) ) OR ("glucose metabolism"[All Fields])) OR ("glucose level"[All Fields])) OR ("fasting glucose"[All Fields])) OR ("fasting insulin"[All Fields])) OR ("insulin sensitivity"[All Fields])) OR ("hemoglobin a1c"[All Fields])) OR ("postprandial glucose"[All Fields])) OR ("insulin response"[All Fields])) OR ("body composition"[All Fields])) OR ("body fat"[All Fields])) OR ("body constitution"[All Fields])) OR ("bowel function"[All Fields])) OR ("bowel functions"[All Fields])) OR ("bowel dysfunction"[All Fields])) OR ("colon function"[All Fields])) OR ("colon functions"[All Fields])) OR (stool)) OR ("transit time"[All Fields])) OR ("intestinal function"[All Fields])) OR ("intestinal functions"[All Fields])) OR ("bowel movements"[All Fields])) OR ("bowel movement"[All Fields]))) AND ("Diet"[Mesh] OR "Feeding Behavior"[Mesh] OR "Food Preferences"[Mesh] OR "Eating"[Mesh:NoExp] OR diet[All Fields] OR "food choice"[All Fields] OR "food choices"[All Fields] OR intake[All Fields] OR intakes[All Fields] OR "dietary habits"[All Fields] OR "dietary habit"[All Fields] OR "dietary pattern"[All Fields] OR "dietary patterns"[All Fields] OR "eating pattern"[All Fields] OR "eating patterns"[All Fields] OR "food pattern"[All Fields] OR "food patterns"[All Fields] OR "feeding behavior"[All Fields] OR "diet habits"[All Fields] OR "diet habit"[All Fields] OR "food preference"[All Fields] OR eating[All Fields] OR consumption[All Fields] OR feeding[All Fields])) AND (((((((((((sugar*[Title/Abstract]) OR ("dietary sucrose"[Title/Abstract]) OR ("Dietary Sucrose"[Mesh])) OR ("High Fructose Corn Syrup"[Mesh] OR "isoglucose"[Title/Abstract] OR "sweetening"[Title/Abstract] OR sweetener*[Title/Abstract] OR "sugar sweetened beverages"[Title/Abstract] OR "sugar sweetened beverage"[Title/Abstract] OR glucose fructose[Title/Abstract] OR "Sweetening Agents"[Mesh])) OR (((((galactose[MeSH Terms]) OR (galactose[Title/Abstract])) OR (galactose[MeSH Terms])) OR (glucose[Title/Abstract])) OR (dextrose[Title/Abstract]))) OR ((((("Polysaccharides"[Mesh:NoExp]) OR "Fructans"[Mesh]) OR "Inulin"[Mesh]) OR "Glucans"[Mesh]) OR (((((((((polysaccharide*[Title/Abstract]) OR (saccharose[Title/Abstract])) OR (glycan*[Title/Abstract])) OR (fructan*[Title/Abstract])) OR (levans[Title/Abstract])) OR (inulin[Title/Abstract])) OR (glucan*[Title/Abstract])) OR (polyglucose*[Title/Abstract])) OR (polycose[Title/Abstract])))) OR (((((oligosaccharide*[Title/Abstract]) OR (oligofructose[Title/Abstract])) OR (fructooligosaccharide[Title/Abstract])) OR (honey[Title/Abstract])) OR (("Oligosaccharides"[Mesh:NoExp]) OR "Honey"[Mesh]))) OR (("Monosaccharides"[Mesh:NoExp]) OR (monosaccharide*[Title/Abstract]))) OR (("high fructose corn"[Title/Abstract] OR "glucose fructose"[Title/Abstract] OR maize[Title/Abstract] OR corn[Title/Abstract]) AND (syrup[Title/Abstract]))) OR (disaccharide*[Title/Abstract] OR disaccharose[Title/Abstract] OR saccharobiose[Title/Abstract] OR cellobiose[Title/Abstract] OR isomaltose[Title/Abstract] OR lactose[Title/Abstract] OR maltose[Title/Abstract] OR melibiose[Title/Abstract] OR trehalose[Title/Abstract] OR isomaltulose[Title/Abstract] OR palatinose[Title/Abstract] OR psicose[Title/Abstract] OR allulose[Title/Abstract])) OR ("Dietary Carbohydrates"[Mesh] OR carbohydrate*[Title/Abstract] OR "High Fructose Corn Syrup"[Mesh])) OR ("Disaccharides"[Mesh] OR "Cellobiose"[Mesh] OR "Isomaltose"[Mesh] OR "Lactose"[Mesh] OR "Maltose"[Mesh] OR "Melibiose"[Mesh] OR "Trehalose"[Mesh] OR "isomaltulose" [Supplementary Concept]) OR "psicose" [Supplementary Concept]))) AND ((((((((((((randomized controlled trial[Publication Type] OR controlled clinical trial[Publication Type] OR placebo[Title/Abstract] OR randomized[Title/Abstract] OR randomly[Title/Abstract] OR trial[Title/Abstract] OR groups[Title/Abstract]) OR ("Epidemiologic Studies"[Mesh:NoExp])) OR ("Cohort Studies"[Mesh])) OR ((cohort*[Title/Abstract]) AND ((study[Title/Abstract]) OR (studies[Title/Abstract]) OR (trial[Title/Abstract])))) OR (Cohort analy*[Title/Abstract])) OR (("follow up"[Title/Abstract]) AND ((study[Title/Abstract]) OR (studies[Title/Abstract]) OR (trial[Title/Abstract])))) OR ("Longitudinal Studies"[Mesh] OR "Prospective Studies"[Mesh] OR "Follow-Up Studies"[Mesh])) OR (observational[Title/Abstract] AND ((study[Title/Abstract]) OR (studies[Title/Abstract]) OR (trial[Title/Abstract])))) OR (epidemiologic[Title/Abstract] AND ((study[Title/Abstract]) OR (studies[Title/Abstract]) OR (trial[Title/Abstract])))) OR (longitudinal[Title/Abstract] AND ((study[Title/Abstract]) OR (studies[Title/Abstract]) OR (trial[Title/Abstract])))) OR (prospective*[Title/Abstract] AND ((study[Title/Abstract]) OR (studies[Title/Abstract]) OR (trial[Title/Abstract])))) NOT ((animals[MeSH Terms]) NOT (humans[MeSH Terms])))) AND ("Child"[Mesh] OR "Infant"[Mesh] OR child*[Title/Abstract] OR childhood[Title/Abstract] OR infant*[Title/Abstract] OR infancy[Title/Abstract] OR toddler*[Title/Abstract] OR babies[Title/Abstract] OR babies[Title/Abstract] OR preschooler*[Title/Abstract] OR "preschool-aged"[Title/Abstract]) |
| --- |

**Table S2a. MMAT assessment of risk of bias of observational studies**

| Study ID |  | S1. Are there clear research questions? | S2. Do the collected data allow to address the research questions? | 3.1. Are the participants representative of the target population? | 3.2. Are measurements appropriate regarding both the outcome and intervention (or exposure)? | 3.3. Are there complete outcome data? | 3.4. Are the confounders accounted for in the design and analysis? | 3.5. During the study period, is the intervention administered (or exposure occurred) as intended? |
| --- | --- | --- | --- | --- | --- | --- | --- | --- |
| 23 | Beyerlein et al. (2015) | + | + | + | + | + | + | + |
| 24 | Buyken et al. (2008) | + | + | - | ? | + | + | + |
| 25 | Byrne et al. (2018) | + | + | - | - | + | - | + |
| 26 | Cantoral et al. (2016) | + | + | - | + | + | + | + |
| 27 | Chaidez et al. (2013) | + | + | - | - | - | + | + |
| 28 | Cowin et al. (2001) | + | + | + | + | ? | + | + |
| 29 | DeBoer et al. (2013) | + | + | + | + | + | ? | + |
| 30 | Dubois et al. (2007) | + | + | + | - | + | + | + |
| 31 | Garden et al. (2011) | + | + | + | + | - | + | + |
| 32 | Herbst et al. (2011) | + | + | - | ? | - | + | + |
| 33 | Huus et al. (2008) | + | + | + | ? | + | ? | + |
| 34 | Jardi et al. (2019) | + | + | - | - | + | + | + |
| 35 | Kiefte-de Jong et al. (2013) | + | + | + | - | ? | - | ? |
| 36 | Leermakers et al. (2015b) | + | + | + | + | + | + | + |
| 37 | Leermakers et al. (2015a) | + | + | + | + | + | + | + |
| 38 | Lim et al. (2008) | + | + | + | + | - | + | + |
| 39 | Newby et al. (2003) | + | + | - | + | - | + | + |
| 40 | Newby et al. (2004) | + | + | - | + | - | + | + |
| 41 | Nguyen et al. (2020) | + | + | + | + | ? | + | + |
| 42 | Quah et al. (2019) | + | + | ? | - | + | + | + |
| 43 | Scaglioni et al. (2000) | + | + | ? | + | + | + | ? |
| 44 | Shefferly et al. (2016) | + | + | + | - | + | + | + |
| 45 | Sonneville et al. (2015) | + | + | + | - | + | + | + |
| 46 | Tappin et al. (2020) | + | + | + | - | + | + | + |
| 47 | Taylor et al. (2016) | + | + | + | - | + | - | + |
| 48 | Van Gijssel et al. (2016) | + | + | + | + | ? | + | + |
| 49 | Warner et al. (2006) | + | + | + | - | + | + | + |
| 50 | Williams et al. (2008) | + | + | + | ? | - | + | + |
| 51 | Wu et al. (2021) | + | + | + | - | + | + | + |

+ yes, - no, ? can’t tell

**Table S2b. MMAT assessment of risk of bias of Randomized Controlled Trials**

| Study ID | | S1. Are there clear research questions? | S2. Do the collected data allow to address the research questions? | 2.1. Is randomization appropriately performed? | 2.2. Are the groups comparable at baseline? | 2.3. Are there complete outcome data? | 2.4. Are outcome assessors blinded to the intervention provided? | 2.5 Did the participants adhere to the assigned intervention? |
| --- | --- | --- | --- | --- | --- | --- | --- | --- |
| 52 | Nakamura et al. (2006) | + | + | + | + | + | + | ? |
| 53 | Waligora-Dupriet et al. (2007) | + | + | ? | + | - | ? | + |

+ yes, - no, ? can’t tell

**Table S3. Excluded studies table**

| **Supplementary reference number** | **Reason for exclusion** |
| --- | --- |
| 1-43 | Population |
| 44-63 | Intervention; Exposure timing/lack of dietary exposure of interest |
| 64-76 | Irrelevant outcome of interest |
| 77-132 | Study design (studies other than prospective cohorts, randomized controlled trials) |
| 133-153 | Only abstract available (no full-text available) |

**References:**

S1. Alexy U, Sichert-Hellert W, Kersting M, Manz F, Schöch G. Fruit juice consumption and the prevalence of obesity and short stature in german preschool children: results of the DONALD Study. Dortmund Nutritional and Anthropometrical Longitudinally Designed. Journal of pediatric gastroenterology and nutrition. 1999;29(3):343-349. doi:10.1097/00005176-199909000-00019

S2. Ayonrinde OT, Oddy WH, Adams LA, et al. Infant nutrition and maternal obesity influence the risk of non-alcoholic fatty liver disease in adolescents. Journal of hepatology. 2017;67(3):568-576. doi:10.1016/J.JHEP.2017.03.029

S3. Brauchla M, Juan W, Story J, Kranz S. Sources of Dietary Fiber and the Association of Fiber Intake with Childhood Obesity Risk (in 2-18 Year Olds) and Diabetes Risk of Adolescents 12-18 Year Olds: NHANES 2003-2006. Journal of nutrition and metabolism. 2012;2012doi:10.1155/2012/736258

S4. Brown CM, Dulloo AG, Montani JP. Sugary drinks in the pathogenesis of obesity and cardiovascular diseases. International journal of obesity (2005). 2008;32 Suppl 6:S28-S34. doi:10.1038/IJO.2008.204

S5. Evans RA, Frese M, Romero J, Cunningham JH, Mills KE. Fructose replacement of glucose or sucrose in food or beverages lowers postprandial glucose and insulin without raising triglycerides: A systematic review and meta-analysis. American Journal of Clinical Nutrition. 2017;106(2):506-518. doi:10.3945/ajcn.116145151

S6. Evans RA, Frese M, Romero J, Cunningham JH, Mills KE. Chronic fructose substitution for glucose or sucrose in food or beverages has little effect on fasting blood glucose, insulin, or triglycerides: A systematic review and meta-analysis. American Journal of Clinical Nutrition. 2017;106(2):519-529. doi:10.3945/ajcn.116145169

S7. Flogan C, Dahl W. Effects of Fiber-Fortified Foods on Children With Constipation: Potential Improved Stool Frequency and Decreased Energy Intake. ICAN: Infant, Child, & Adolescent Nutrition. 2010;2(5):312-317. doi:10.1177/1941406410383980

S8. Hampson HE, Jones RB, Berger PK, et al. Adverse Effects of Infant Formula Made with Corn-Syrup Solids on the Development of Eating Behaviors in Hispanic Children. Nutrients. 2022;14(5)doi:10.3390/nu14051115

S9. Hoekstra JH, Szajewska H, Zikri MA, et al. Oral rehydration solution containing a mixture of non-digestible carbohydrates in the treatment of acute diarrhea: a multicenter randomized placebo controlled study on behalf of the ESPGHAN working group on intestinal infections. Journal of pediatric gastroenterology and nutrition. 2004;39(3):239-245. doi:10.1097/00005176-200409000-00003

S10. Hui LL, Nelson EAS. Meal glycaemic load of normal-weight and overweight Hong Kong children. European journal of clinical nutrition. 2006;60(2):220-227. doi:10.1038/SJ.EJCN.1602305

S11. Jensen BW, Nichols M, Allender S, et al. Inconsistent associations between sweet drink intake and 2-year change in BMI among Victorian children and adolescents. Pediatric obesity. 2013;8(4):271-283. doi:10.1111/j.2047-6310.2013.00174.x

S12. Jones TW, Borg WP, Boulware SD, McCarthy G, Sherwin RS, Tamborlane WV. Enhanced adrenomedullary response and increased susceptibility to neuroglycopenia: mechanisms underlying the adverse effects of sugar ingestion in healthy children. The Journal of pediatrics. 1995;126(2):171-177. doi:10.1016/s0022-3476(95)70541-4

S13. Katan MB, De Ruyter JC, Kuijper LDJ, Chow CC, Hall KD, Olthof MR. Impact of masked replacement of sugar-sweetened with sugar-free beverages on body weight increases with initial bmi: Secondary analysis of data from an 18 month double-blind trial in children. PloS one. 2016;11(7)doi:10.1371/journal.pone.0159771

S14. Larrosa S, Luque V, Grote V, et al. Fibre Intake Is Associated with Cardiovascular Health in European Children. Nutrients. 2020;13(1)doi:10.3390/nu13010012

S15. Lebenthal E, Khin Maung U, Khin Myat T, et al. High-calorie, rice-derived, short-chain, glucose polymer-based oral rehydration solution in acute watery diarrhea. Acta paediatrica. 1995;84(2):165-172. doi:10.1111/j.1651-2227.1995.tb13603.x

S16. Lee JJ, Brett NR, Wong VCH, Totosy de Zepetnek JO, Fiocco AJ, Bellissimo N. Effect of potatoes and other carbohydrate-containing foods on cognitive performance, glycemic response, and satiety in children. Applied physiology, nutrition, and metabolism = Physiologie appliquee, nutrition et metabolisme. 2019;44(9):1012-1019. doi:10.1139/apnm-2018-0792

S17. Lohner S, Jakobik V, Mihályi K, et al. Inulin-type fructan supplementation of 3- To 6-year-old children is associated with higher fecal Bifidobacterium concentrations and fewer febrile episodes requiring medical attention. Journal of Nutrition. 2018;148(8):1300-1308. doi:10.1093/jn/nxy120

S18. Ludwig DS, Majzoub JA, Al-Zahrani A, Dallal GE, Blanco I, Roberts SB. High glycemic index foods, overeating, and obesity. Pediatrics. 1999;103(3)doi:10.1542/PEDS.103.3.E26

S19. Macgillivray S, Fahey T, McGuire W. Lactose avoidance for young children with acute diarrhoea. Cochrane Database of Systematic Reviews. 2013;2013(10)doi:10.1002/14651858.CD005433.pub2

S20. Marshall TA, Curtis AM, Cavanaugh JE, Warren JJ, Levy SM. Child and Adolescent Sugar-Sweetened Beverage Intakes Are Longitudinally Associated with Higher Body Mass Index z Scores in a Birth Cohort Followed 17 Years. Journal of the Academy of Nutrition and Dietetics. 2019;119(3):425-434. doi:10.1016/j.jand.2018.11.003

S21. Mikkilä V, Räsänen L, Raitakari OT, Pietinen P, Viikari J. Consistent dietary patterns identified from childhood to adulthood: the cardiovascular risk in Young Finns Study. The British journal of nutrition. 2005;93(6):923-931. doi:10.1079/BJN20051418

S22. Morrison JA, Glueck CJ, Woo JG, Wang P. Risk factors for cardiovascular disease and type 2 diabetes retained from childhood to adulthood predict adult outcomes: the Princeton LRC Follow-up Study. International journal of pediatric endocrinology. 2012;2012(1)doi:10.1186/1687-9856-2012-6

S23. Nielsen BM, Bjørnsbo KS, Tetens I, Heitmann BL. Dietary glycaemic index and glycaemic load in Danish children in relation to body fatness. The British journal of nutrition. 2005;94(6):992-997. doi:10.1079/BJN20051465

S24. Park S, Pan L, Sherry B, Li R. The association of sugar-sweetened beverage intake during infancy with sugar-sweetened beverage intake at 6 years of age. Pediatrics. 2014;134 Suppl 1(Suppl 1):S56-62. doi:10.1542/peds.2014-0646J

S25. Rizkalla SW. Health implications of fructose consumption: A review of recent data. Nutrition and Metabolism. 2014;7(1)doi:10.1186/1743-7075-7-82

S26. Robinson SL, Sundaram R, Putnick DL, et al. Predictors of Age at Juice Introduction and Associations with Subsequent Beverage Intake in Early and Middle Childhood. The Journal of nutrition. 2021;151(11):3516-3523. doi:10.1093/jn/nxab260

S27. Rompay MIV, McKeown NM, Goodman E, et al. Sugar-Sweetened Beverage Intake Is Positively Associated with Baseline Triglyceride Concentrations, and Changes in Intake Are Inversely Associated with Changes in HDL Cholesterol over 12 Months in a Multi-Ethnic Sample of Children. The Journal of nutrition. 2015;145(10):2389-2395. doi:10.3945/JN.115.212662

S28. Roshon MS, Hagen RL. Sugar consumption, locomotion, task orientation, and learning in preschool children. Journal of abnormal child psychology. 1989;17(3):349-357.

S29. Ruottinen S, Niinikoski H, Lagström H, et al. High sucrose intake is associated with poor quality of diet and growth between 13 months and 9 years of age: The special turku coronary risk factor intervention project. Pediatrics. 2008;121(6):e1676-e1685. doi:10.1542/peds.2007-1642

S30. Saneian H, Yaghini O, Modaresi M, Razmkhah N. Lactose-free compared with lactose-containing formula in dietary management of acute childhood diarrhea. Iranian journal of pediatrics. 2012;22(1):82-86.

S31. Scaglioni S, Stival G, Giovannini M. Dietary glycemic load, overall glycemic index, and serum insulin concentrations in healthy schoolchildren. The American journal of clinical nutrition. 2004;79(2):339-340. doi:10.1093/AJCN/79.2.339

S32. Simakachorn N, Tongpenyai Y, Tongtan O, Varavithya W. Randomized, double-blind clinical trial of a lactose-free and a lactose-containing formula in dietary management of acute childhood diarrhea. Journal of the Medical Association of Thailand. 2004;87(6):641-649.

S33. Soldi S, Vasileiadis S, Lohner S, et al. Prebiotic supplementation over a cold season and during antibiotic treatment specifically modulates the gut microbiota composition of 3-6 year-old children. Beneficial Microbes. 2019;10(3):253-263. doi:10.3920/BM2018.0116 34. Starc TJ, Shea S, Cohn LC, Mosca L, Gersony WM, Deckelbaum RJ. Greater dietary intake of simple carbohydrate is associated with lower concentrations of high-density-lipoprotein cholesterol in hypercholesterolemic children. The American journal of clinical nutrition. 1998;67(6):1147-1154. doi:10.1093/AJCN/67.6.1147

S35. Toporovski MS, de Morais MB, Abuhab A, Crippa Júnior MA. Effect of Polydextrose/Fructooligosaccharide Mixture on Constipation Symptoms in Children Aged 4 to 8 Years. Nutrients. 2021;13(5)doi:10.3390/nu13051634

S36. Tuohy KM, Kolida S, Lustenberger AM, Gibson GR. The prebiotic effects of biscuits containing partially hydrolysed guar gum and fructo-oligosaccharides--a human volunteer study. The British journal of nutrition. 2001;86(3):341-348. doi:10.1079/BJN2001394

S37. Wall C, Todaro W, Edwards K, Cleghorn G. A 3-hour quantitative comparison of glucose-based versus rice-based oral rehydration solution intake by children with diarrhoea in Port Moresby General Hospital. Papua and New Guinea medical journal. 1995;38(4):284-286.

S38. Wall CR, Swanson CE, Cleghorn GJ. A controlled trial comparing the efficacy of rice-based and hypotonic glucose oral rehydration solutions in infants and young children with gastroenteritis. Journal of Gastroenterology and Hepatology. 1997;12(1):24-28. doi:10.1111/j.1440-1746.1997.tb00340.x

S39. Williams CL, Strobino BA, Bollella M, Brotanek J. Cardiovascular risk reduction in preschool children: the "Healthy Start" project. Journal of the American College of Nutrition. 2004;23(2):117-123. doi:10.1080/07315724.2004.10719351

S40. Wolraich ML, Lindgren SD, Stumbo PJ, Stegink LD, Appelbaum MI, Kiritsy MC. Effects of diets high in sucrose or aspartame on the behavior and cognitive performance of children. New England Journal of Medicine. 1994;330(5):301-307. doi:10.1056/NEJM199402033300501

S41. Yu CJ, Du JC, Chiou HC, et al. Sugar-Sweetened Beverage Consumption Is Adversely Associated with Childhood Attention Deficit/Hyperactivity Disorder. International journal of environmental research and public health. 2016;13(7)doi:10.3390/IJERPH13070678

S42. Yu P, Chen Y, Zhao A, et al. Consumption of sugar-sweetened beverages and its association with overweight among young children from China. Public health nutrition. 2016;19(13):2336-2346. doi:10.1017/S1368980016001373

S43. Zheng M, Rangan A, Allman-Farinelli M, Rohde JF, Olsen NJ, Heitmann BL. Replacing sugary drinks with milk is inversely associated with weight gain among young obesity-predisposed children. British Journal of Nutrition. 2015;114(9):1448-1455. doi:10.1017/S0007114515002974

S44. Barclay DV, Gil-Ramos J, Mora JO, Dirren H. A packaged rice-based oral rehydration solution for acute diarrhea. Journal of pediatric gastroenterology and nutrition. 1995;20(4):408-416. doi:10.1097/00005176-199505000-00006

S45. Heron J, Grzeda M, Tappin D, Von Gontard A, Joinson C. Early childhood risk factors for constipation and soiling at school age: an observational cohort study. BMJ paediatrics open. 2018;2(1)doi:10.1136/BMJPO-2017-000230

S46. Reilly JJ, Armstrong J, Dorosty AR, et al. Early life risk factors for obesity in childhood: cohort study. BMJ (Clinical research ed). 2005;330(7504):1357-1359. doi:10.1136/BMJ.38470.670903.E0

S47. Williams CL, Bollella MC, Strobino BA, Boccia L, Campanaro L. Plant stanol ester and bran fiber in childhood: effects on lipids, stool weight and stool frequency in preschool children. Journal of the American College of Nutrition. 1999;18(6):572-581. doi:10.1080/07315724.1999.10718891

S48. Williams CL, Bollella MC, Strobino BA, et al. "Healthy-start": outcome of an intervention to promote a heart healthy diet in preschool children. Journal of the American College of Nutrition. 2002;21(1):62-71. doi:10.1080/07315724.2002.10719195

S49. Aumueller N, Gruszfeld D, Gradowska K, et al. Associations of sugar intake with anthropometrics in children from ages 2 until 8 years in the EU Childhood Obesity Project. European journal of nutrition. 2020;59(6):2593-2601. doi:10.1007/s00394-019-02107-0

S50. Bell LK, Schammer C, Devenish G, et al. Dietary patterns and risk of obesity and early childhood caries in Australian toddlers: Findings from an australian cohort study. Nutrients. 2019;11(11)doi:10.3390/nu11112828

S51. Beyerlein A, Uusitalo UM, Virtanen SM, et al. Intake of Energy and Protein is Associated with Overweight Risk at Age 5.5 Years: Results from the Prospective TEDDY Study. Obesity. 2017;25(8):1435-1441. doi:10.1002/oby.21897

S52. Cowin I, Emmett P. Cholesterol and triglyceride concentrations, birthweight and central obesity in pre-school children. ALSPAC Study Team. Avon Longitudinal Study of Pregnancy and Childhood. International journal of obesity and related metabolic disorders : journal of the International Association for the Study of Obesity. 2000;24(3):330-339. doi:10.1038/SJ.IJO.0801133

S53. Flores G, Lin H. Factors predicting severe childhood obesity in kindergarteners. International Journal of Obesity. 2013;37(1):31-39. doi:10.1038/ijo.2012.168

S54. Jansen PW, Tharner A, Van Der Ende J, et al. Feeding practices and child weight: is the association bidirectional in preschool children? The American journal of clinical nutrition. 2014;100(5):1329-1336. doi:10.3945/AJCN.114.088922

S55. Larrosa S, Luque V, Grote V, et al. Fibre intake is associated with cardiovascular health in european children. Nutrients. 2021;13(1):1-14. doi:10.3390/nu13010012

S56. LeCroy MN, Bryant M, Albrecht SS, et al. Obesogenic home food availability, diet, and BMI in Pakistani and White toddlers. Maternal and Child Nutrition. 2021;17(3)doi:10.1111/mcn.13138

S57. Nguyen T, Sokal-Gutierrez K, Lahiff M, Fernald L, Ivey SL. Early childhood factors associated with obesity at age 8 in Vietnamese children: The Young Lives Cohort Study. BMC public health. 2021;21(1):301. doi:10.1186/s12889-021-10292-z

S58. Niinikoski H, Ruottinen S. Is carbohydrate intake in the first years of life related to future risk of NCDs? Nutrition, Metabolism and Cardiovascular Diseases. 2012;22(10):770-774. doi:10.1016/j.numecd.2012.05.002

S59. O'Connor TM, Yang S-J, Nicklas TA. Beverage intake among preschool children and its effect on weight status. Pediatrics. 2006;118(4):e1010-8. doi:10.1542/peds.2005-2348

S60. Pimpin L, Jebb S, Johnson L, Wardle J, Ambrosini GL. Dietary protein intake is associated with body mass index and weight up to 5 y of age in a prospective cohort of twins. American Journal of Clinical Nutrition. 2016;103(2):389-397. doi:10.3945/ajcn.115.118612

S61. Sirkka O, Fleischmann M, Abrahamse-Berkeveld M, et al. Dietary patterns in early childhood and the risk of childhood overweight: The gecko drenthe birth cohort. Nutrients. 2021;13(6)doi:10.3390/nu13062046

S62. Skinner JD, Bounds W, Carruth BR, Morris M, Ziegler P. Predictors of children's body mass index: A longitudinal study of diet and growth in children aged 2-8y. International Journal of Obesity. 2004;28(4):476-482. doi:10.1038/sj.ijo.0802405

S63. Williams CL, Strobino B, Bollella M, Brotanek J. Body size and cardiovascular risk factors in a preschool population. Preventive cardiology. 2004;7(3):116-121. doi:10.1111/J.1520-037X.2004.03224.X

S64. Bennett CA, de Silva-Sanigorski AM, Nichols M, Bell AC, Swinburn BA. Assessing the intake of obesity-related foods and beverages in young children: comparison of a simple population survey with 24 hr-recall. The international journal of behavioral nutrition and physical activity. 2009;6doi:10.1186/1479-5868-6-71

S65. Bjelland M, Brantsæter AL, Haugen M, Meltzer HM, Nystad W, Andersen LF. Changes and tracking of fruit, vegetables and sugar-sweetened beverages intake from 18 months to 7 years in the Norwegian Mother and Child Cohort Study. BMC public health. 2013;13:793. doi:10.1186/1471-2458-13-793

S66. Cuello-Garcia C, Fiocchi A, Pawankar R, et al. Prebiotics for the prevention of allergies: A systematic review and meta-analysis of randomized controlled trials. Clinical and experimental allergy : journal of the British Society for Allergy and Clinical Immunology. 2017;47(11):1468-1477. doi:10.1111/cea.13042

S67. Northstone K, Emmett P. The associations between feeding difficulties and behaviours and dietary patterns at 2 years of age: the ALSPAC cohort. Maternal & child nutrition. 2013;9(4):533-542. doi:10.1111/J.1740-8709.2012.00399.X

S68. Padilha LL, Vianna EO, Vale ATM, Nascimento JXPT, da Silva AAM, Ribeiro CCC. Pathways in the association between sugar sweetened beverages and child asthma traits in the 2nd year of life: Findings from the BRISA cohort. Pediatric Allergy and Immunology. 2020;31(5):480-488. doi:10.1111/pai.13243

S69. Ruottinen S, Lagström HK, Niinikoski H, et al. Dietary fiber does not displace energy but is associated with decreased serum cholesterol concentrations in healthy children. The American journal of clinical nutrition. 2010;91(3):651-661. doi:10.3945/ajcn.2009.28461

S70. Wright LS, Rifas-Shiman SL, Oken E, Litonjua AA, Gold DR. Prenatal and Early Life Fructose, Fructose-Containing Beverages, and Midchildhood Asthma. Annals of the American Thoracic Society. 2018;15(2):217-224. doi:10.1513/AnnalsATS.201707-530OC

S71. Geurtsen ML, Santos S, Gaillard R, Felix JF, Jaddoe VWV. Associations Between Intake of Sugar-Containing Beverages in Infancy With Liver Fat Accumulation at School Age. Hepatology. 2021;73(2):560-570. doi:10.1002/hep.31611

S72. Thurber KA, Dobbins T, Neeman T, Banwell C, Banks E. Body mass index trajectories of Indigenous Australian children and relation to screen time, diet, and demographic factors. Obesity (Silver Spring). Apr 2017;25(4):747-756. doi:10.1002/oby.21783

S73. Garden FL, Marks GB, Simpson JM, Webb KL. Body mass index (BMI) trajectories from birth to 11.5 years: relation to early life food intake. Nutrients. 2012;4(10):1382-98. doi:10.3390/nu4101382

S74. Hwang IT, Ju YS, Lee HJ, Shim YS, Jeong HR, Kang MJ. Body mass index trajectories and adiposity rebound during the first 6 years in Korean children: Based on the National Health Information Database, 2008-2015. PloS one. 2020;15(10):e0232810. doi:10.1371/journal.pone.0232810

S75. Dorosty AR, Emmett PM, Cowin S, Reilly JJ. Factors associated with early adiposity rebound. ALSPAC Study Team. Pediatrics. 2000;105(5):1115-8. doi:10.1542/peds.105.5.1115

S76. Faith MS, Dennison BA, Edmunds LS, Stratton HH. Fruit juice intake predicts increased adiposity gain in children from low-income families: weight status-by-environment interaction. Pediatrics. 2006;118(5):2066-75. doi: 10.1542/peds.2006-1117.

S77. Aggett PJ, Agostoni C, Axelsson I, et al. Nondigestible carbohydrates in the diets of infants and young children: a commentary by the ESPGHAN Committee on Nutrition. Journal of pediatric gastroenterology and nutrition. 2003;36(3):329-337. doi:10.1097/00005176-200303000-00006

S78. Boyd A, Golding J, Macleod J, et al. Cohort Profile: the 'children of the 90s'--the index offspring of the Avon Longitudinal Study of Parents and Children. International journal of epidemiology. 2013;42(1):111-127. doi:10.1093/IJE/DYS064

S79. Chumpitazi BP, Shulman RJ. Dietary Carbohydrates and Childhood Functional Abdominal Pain. Annals of Nutrition and Metabolism. 2016;68(1):8-17. doi:10.1159/000445390

S80. Dereń K, Weghuber D, Caroli M, et al. Consumption of Sugar-Sweetened Beverages in Paediatric Age: A Position Paper of the European Academy of Paediatrics and the European Childhood Obesity Group. Annals of Nutrition and Metabolism. 2019;74(4):296-302. doi:10.1159/000499828

S81. Edwards CA, Parrett AM. Dietary fibre in infancy and childhood. The Proceedings of the Nutrition Society. 2003;62(1):17-23. doi:10.1079/PNS2002231

S82. Edwards CA, Xie C, Garcia AL. Dietary fibre and health in children and adolescents. The Proceedings of the Nutrition Society. 2015;74(3):292-302. doi:10.1017/S0029665115002335

S83. Emmett P. Dietary assessment in the Avon Longitudinal Study of Parents and Children. European journal of clinical nutrition. 2009;63 Suppl 1:S38-S44. doi:10.1038/EJCN.2008.63

S84. Garnett BR, Rosenberg KD, Morris DS. Consumption of soda and other sugar-sweetened beverages by 2-year-olds: findings from a population-based survey. Public health nutrition. 2013;16(10):1760-1767. doi:10.1017/S1368980012004399

S85. Grimes CA, Riddell LJ, Campbell KJ, Nowson CA. Dietary salt intake, sugar-sweetened beverage consumption, and obesity risk. Pediatrics. 2013;131(1):14-21. doi:10.1542/PEDS.2012-1628

S86. Ha K, Chung S, Lee HS, et al. Association of Dietary Sugars and Sugar-Sweetened Beverage Intake with Obesity in Korean Children and Adolescents. Nutrients. 2016;8(1)doi:10.3390/NU8010031

S87. Hu FB. Resolved: There is sufficient scientific evidence that decreasing sugar-sweetened beverage consumption will reduce the prevalence of obesity and obesity-related diseases. Obesity Reviews. 2013;14(8):606-619. doi:10.1111/obr.12040

S88. Isolauri E, Rautava S, Salminen S, Collado MC. Early-Life Nutrition and Microbiome Development. Nestle Nutrition Institute workshop series. 2019;90:151-162. doi:10.1159/000490302

S89. Kranz S, Brauchla M, Slavin JL, Miller KB. What do we know about dietary fiber intake in children and health? The effects of fiber intake on constipation, obesity, and diabetes in children. Advances in nutrition. 2012;3(1):47-53. doi:10.3945/AN.111.001362

S90. Morais MB, Vítolo MR, Aguirre ANC, Fagundes-Neto U. Measurement of low dietary fiber intake as a risk factor for chronic constipation in children. Journal of pediatric gastroenterology and nutrition. 1999;29(2):132-135. doi:10.1097/00005176-199908000-00007

S91. Ooi L-G, Liong M-T. Cholesterol-lowering effects of probiotics and prebiotics: a review of in vivo and in vitro findings. International journal of molecular sciences. 2010;11(6):2499-2522. doi:10.3390/ijms11062499

S92. Pérez-Farinós N, Villar-Villalba C, López Sobaler AM, et al. The relationship between hours of sleep, screen time and frequency of food and drink consumption in Spain in the 2011 and 2013 ALADINO: a cross-sectional study. BMC public health. 2017;17(1):1-12. doi:10.1186/S12889-016-3962-4

S93. Stricker S, Rudloff S, Geier A, Steveling A, Roeb E, Zimmer K-P. Fructose Consumption-Free Sugars and Their Health Effects. Deutsches Arzteblatt international. 2021;118(5):71-78. doi:10.3238/arztebl.m2021.0010

S94. Vos MB, Kaar JL, Welsh JA, et al. Added sugars and cardiovascular disease risk in children: A scientific statement from the American Heart Association. Circulation. 2017;135(19):e1017-e1034. doi:10.1161/CIR.0000000000000439

S95. Wang YC, Bleich SN, Gortmaker SL. Increasing caloric contribution from sugar-sweetened beverages and 100% fruit juices among US children and adolescents, 1988-2004. Pediatrics. 2008;121(6)doi:10.1542/PEDS.2007-2834

S96. Alamri E, Bayomy H, Mohammedsaledh Z. The role of gut microbiome in obesity: A systematic review. Proceedings of the Nutrition Society. 2022;81(OCE1):E33. doi:10.1017/S0029665122000337

S97. Auerbach BJ, Dibey S, Vallila-Buchman P, Kratz M, Krieger J. Review of 100% Fruit Juice and Chronic Health Conditions: Implications for Sugar-Sweetened Beverage Policy. Advances in nutrition. 2018;9(2):78-85. doi:10.1093/advances/nmx006

S98. Avery A, Bostock L, McCullough F. A systematic review investigating interventions that can help reduce consumption of sugar-sweetened beverages in children leading to changes in body fatness. Journal of human nutrition and dietetics : the official journal of the British Dietetic Association. 2015;28 Suppl 1(Suppl 1):52-64. doi:10.1111/jhn.12267

S99. Bucher Della Torre S, Keller A, Laure Depeyre J, Kruseman M. Sugar-Sweetened Beverages and Obesity Risk in Children and Adolescents: A Systematic Analysis on How Methodological Quality May Influence Conclusions. Journal of the Academy of Nutrition and Dietetics. 2016;116(4):638-659. doi:10.1016/j.jand.2015.05.020

S100. Chung M, Ma J, Patel K, Berger S, Lau J, Lichtenstein A. Fructose consumption and non-alcoholic fatty liver disease: A systematic review and meta-analysis. Endocrine Reviews. 2013;34(3)

S101. Elia M, Engfer MB, Green CJ, Silk DBA. Systematic review and meta-analysis: the clinical and physiological effects of fibre-containing enteral formulae. Alimentary pharmacology & therapeutics. 2008;27(2):120-145. doi:10.1111/j.1365-2036.2007.03544.x

S102. English LK, Obbagy JE, Wong YP, et al. Types and amounts of complementary foods and beverages consumed and growth, size, and body composition: A systematic review. American Journal of Clinical Nutrition. 2019;109:956S-977S. doi:10.1093/ajcn/nqy281

S103. Forshee RA, Anderson PA, Storey ML. Sugar-sweetened beverages and body mass index in children and adolescents: A meta-analysis. American Journal of Clinical Nutrition. 2008;87(6):1662-1671. doi:10.1093/ajcn/87.6.1662

S104. Frantsve-Hawley J, Bader JD, Welsh JA, Wright JT. A systematic review of the association between consumption of sugar-containing beverages and excess weight gain among children under age 12. Journal of public health dentistry. 2017;77 Suppl 1:S43-S66. doi:10.1111/jphd.12222

S105. Gibson S. Sugar-sweetened soft drinks and obesity: A systematic review of the evidence from observational studies and interventions. Nutrition Research Reviews. 2008;21(2):134-147. doi:10.1017/S0954422408110976

S106. He S, Stein AD. Early-Life Nutrition Interventions and Associated Long-Term Cardiometabolic Outcomes: A Systematic Review and Meta-Analysis of Randomized Controlled Trials. Advances in nutrition (Bethesda, Md). 2021;12(2):461-489. doi:10.1093/advances/nmaa107

S107. Koplin JJ, Kerr JA, Lodge C, et al. Infant and young child feeding interventions targeting overweight and obesity: A narrative review. Obesity reviews : an official journal of the International Association for the Study of Obesity. 2019;20 Suppl 1:31-44. doi:10.1111/obr.12798

S108. Luger M, Lafontan M, Bes-Rastrollo M, Winzer E, Yumuk V, Farpour-Lambert N. Sugar-Sweetened Beverages and Weight Gain in Children and Adults: A Systematic Review from 2013 to 2015 and a Comparison with Previous Studies. Obesity facts. 2017;10(6):674-693. doi:10.1159/000484566

S109. Lukomskyj N, Allman-Farinelli M, Shi Y, Rangan A. Dietary exposures in childhood and adulthood and cardiometabolic outcomes: a systematic scoping review. Journal of human nutrition and dietetics : the official journal of the British Dietetic Association. 2021;34(3):511-523. doi:10.1111/jhn.12841

S110. Malik VS, Pan A, Willett WC, Hu FB. Sugar-sweetened beverages and weight gain in children and adults: A systematic review and meta-analysis. American Journal of Clinical Nutrition. 2013;98(4):1084-1102. doi:10.3945/ajcn.113.058362

S111. Malik VS, Schulze MB, Hu FB. Intake of sugar-sweetened beverages and weight gain: A systematic review. American Journal of Clinical Nutrition. 2006;84(2):274-288. doi:10.1093/ajcn/84.1.274

S112. Morenga LT, Mallard S, Mann J. Dietary sugars and body weight: Systematic review and meta-analyses of randomised controlled trials and cohort studies. BMJ (Online). 2012;345(7891)doi:10.1136/bmj.e7492

S113. Nissensohn M, Fuentes Lugo D, Serra-Majem L. Sugar-sweetened beverage consumption and obesity in children's meta-analyses: Reaching wrong answers for right questions. Annals of Nutrition and Metabolism. 2017;71:665-666. doi:10.1159/000480486

S114. Nissensohn M, Fuentes Lugo D, Serra-Majem L. Sugar-sweetened beverage consumption and obesity in children's meta-analyses: reaching wrong answers for right questions. Nutricion hospitalaria. 2018;35(2):474-488. doi:10.20960/nh.1492

S115. Noronha JC, Braunstein CR, Blanco Mejia S, et al. The Effect of Small Doses of Fructose and Its Epimers on Glycemic Control: A Systematic Review and Meta-Analysis of Controlled Feeding Trials. Nutrients. 2018;10(11)doi:10.3390/nu10111805

S116. Patro-Gołąb B, Zalewski BM, Kołodziej M, et al. Nutritional interventions or exposures in infants and children aged up to 3 years and their effects on subsequent risk of overweight, obesity and body fat: a systematic review of systematic reviews. Obesity reviews : an official journal of the International Association for the Study of Obesity. 2016;17(12):1245-1257. doi:10.1111/obr.12476

S117. Pérez-Morales E, Bacardí-Gascón M, Jiménez-Cruz A. Sugar-sweetened beverage intake before 6 years of age and weight or BMI status among older children; systematic review of prospective studies. Nutricion hospitalaria. 2013;28(1):47-51.

S118. Reynolds AN, Diep Pham HT, Montez J, Mann J. Dietary fibre intake in childhood or adolescence and subsequent health outcomes: A systematic review of prospective observational studies. Diabetes, Obesity and Metabolism. 2020;22(12):2460-2467. doi:10.1111/dom.14176

S119. Rousham EK, Goudet S, Markey O, et al. Unhealthy Food and Beverage Consumption in Children and Risk of Overweight and Obesity: A Systematic Review and Meta-analysis. Advances in nutrition. 2022;doi:10.1093/advances/nmac032

S120. Schwingshackl L, Hobl LP, Hoffmann G. Effects of low glycaemic index/low glycaemic load vs. high glycaemic index/ high glycaemic load diets on overweight/obesity and associated risk factors in children and adolescents: A systematic review and meta-analysis. Nutrition Journal. 2015;14(1)doi:10.1186/s12937-015-0077-1

S121. Tabbers MM, Boluyt N, Berger MY, Benninga MA. Nonpharmacologic treatments for childhood constipation: Systematic review. Pediatrics. 2011;128(4):753-761. doi:10.1542/peds.2011-0179

S122. Te Morenga L, Mallard S, Mann J. Dietary sugars and body weight: systematic review and meta-analyses of randomised controlled trials and cohort studies. BMJ (Clinical research ed). 2012;346:e7492-e7492. doi:10.1136/bmj.e7492

S123. Trumbo PR, Rivers CR. Systematic review of the evidence for an association between sugar-sweetened beverage consumption and risk of obesity. Nutrition reviews. 2014;72(9):566-574. doi:10.1111/nure.12128

S124. Vercammen KA, Frelier JM, Lowery CM, McGlone ME, Ebbeling CB, Bleich SN. A systematic review of strategies to reduce sugar-sweetened beverage consumption among 0-year to 5-year olds. Obesity Reviews. 2018;19(11):1504-1524. doi:10.1111/obr.12741

S125. Weichert S, Schroten H, Adam R. The role of prebiotics and probiotics in prevention and treatment of childhood infectious diseases. Pediatric Infectious Disease Journal. 2012;31(8):859-862. doi:10.1097/INF.0b013e3182620e52

S126. Williams GM, Tapsell LC, O'Brien CL, Tosh SM, Barrett EM, Beck EJ. Gut microbiome responses to dietary intake of grain-based fibers with the potential to modulate markers of metabolic disease: A systematic literature review. Nutrition reviews. 2021;79(11):1274-1292. doi:10.1093/nutrit/nuaa128

S127. Woodward-Lopez G, Kao J, Ritchie L. To what extent have sweetened beverages contributed to the obesity epidemic? Public health nutrition. 2011;14(3):499-509. doi:10.1017/S1368980010002375

S128. Zalewski BM, Patro B, Veldhorst M, et al. Nutrition of infants and young children (one to three years) and its effect on later health: A systematic review of current recommendations (EarlyNutrition project). Critical reviews in food science and nutrition. 2017;57(3):489-500. doi:10.1080/10408398.2014.888701

S129. Atkin LM, Davies PS. Diet composition and body composition in preschool children. The American journal of clinical nutrition. Jul 2000;72(1):15-21. doi:10.1093/ajcn/72.1.15

S130. Dennison BA, Rockwell HL, Baker SL. Excess fruit juice consumption by preschool-aged children is associated with short stature and obesity. Pediatrics. Jan 1997;99(1):15-22.

S131. Skinner JD, Carruth BR, Moran J, 3rd, Houck K, Coletta F. Fruit juice intake is not related to children's growth. Pediatrics. Jan 1999;103(1):58-64. doi:10.1542/peds.103.1.58

S132. Welsh JA, Cogswell ME, Rogers S, Rockett H, Mei Z, Grummer-Strawn LM. Overweight among low-income preschool children associated with the consumption of sweet drinks: Missouri, 1999-2002. Pediatrics. Feb 2005;115(2):e223-9. doi:10.1542/peds.2004-1148

S133. Nicklas TA, O'Neil CE, Kleinman R. Association between 100% juice consumption and nutrient intake and weight of children aged 2 to 11 years. Archives of pediatrics & adolescent medicine. Jun 2008;162(6):557-65. doi:10.1001/archpedi.162.6.557

S134. Bellissimo N, Poirier KL, Bennett LJ, Lynch N. Effect of sugars-containing beverages on satiety and short-term food intake in normal weight and overweight/obese boys. FASEB Journal. 2011;25

S135. Clemente Yago F, Tapia Collados C, Comino Almenara L, López Peña L, Escrivá Tomás P, González Peraba J. [Lactose-free formula versus adapted formula in acute infantile diarrhea]. Anales espanoles de pediatria. 1993;39(4):309-312.

S136. Elliott EJ, Walker-Smith JA, Farthing MJ, Hunt J, Cameron D. Clinical experience with a hypotonic oral rehydration solution for treatment of pediatric gastroenteritis in the United Kingdom. Clinical therapeutics. 1990;12 Suppl A:86-94.

S137. Fortin-Miller S, Hull H, Carlson S, Colombo J, Kerling E. Added sugar and fructose intake throughout infancy and childhood. Obesity. 2021;29(SUPPL 2):52. doi:10.1002/oby.23329

S138. Kaar J, Woo J, Thompson D, Kalkwarf H, Daniels S, Schmiege S. High prevalence of obesity-related diet and activity behaviors by age 3 identifies need for early interventions. Hormone research in paediatrics. 2020;93(SUPPL 1):11. doi:10.1159/000509566

S139. Lanigan J, Adegboye A, Northstone K, Salisbury C, Singhal A. Nutrition in preschool children and later risk of obesity: A systematic review and meta analysis. Journal of pediatric gastroenterology and nutrition. 2016;62:691-692. doi:10.1097/01.mpg.0000484500.48517.e7

S140. Lohner S, Szili N, Jakobik V, et al. Effect of prebiotic inulin-type fructans on health parameters and intestinal microbiota composition in children aged 3 to 6 years: A randomized, double-blind, placebo-controlled explorative study. Journal of pediatric gastroenterology and nutrition. 2016;62:672. doi:10.1097/01.mpg.0000484500.48517.e7

S141. Lycett K, Juonala M, Lau T, et al. Early clinical markers of overweight/obesity onset and resolution by adolescence: Longitudinal Study of Australian Children. Obesity Research and Clinical Practice. 2019;13(3):253. doi:10.1016/j.orcp.2018.11.048

S142. Lyu LC, Yang YC, Yu HW. Long term follow-up study of refined sugar consumption for children aged 2 to 5 years in Taiwan. Annals of Nutrition and Metabolism. 2013;63:1416. doi:10.1159/000354245

S143. Mohan M, Antony TJ, Malik S, Mathur M. Rice powder oral rehydration solution as an alternative to glucose electrolyte solution. The Indian journal of medical research. 1988;87:234-239.

S144. Nakamura S, Sarker SA, Oku T, Wahed MA, Wagatsuma Y. Effect of daily intake of prebiotic (Fructooligosaccharide) on weight gain and reduction of acute diarrhea among children in a Bangladesh Urban Slum: A randomized double-masked placebo-controlled study. Gastroenterology. 2010;138(5):S51.

S145. Nct. Childcare Healthy Beverage Access, Food and Beverage Intake, and Obesity. 2021;

S146. Ranucci G, Buccigrossi V, Baiardi P, et al. Galacto-oligosaccharide/polydextrose enriched formula prevents respiratory infections and modifies history of all allergy in a popoulation of infants at risk of atopy: The pipa birth cohort study. Journal of pediatric gastroenterology and nutrition. 2017;64:1007. doi:10.1097/01.mpg.0000516381.25680.b4

S147. Ribiero T, Scalabrin D, Pontes M, et al. Effect of a follow-up formula containing prebiotics on constipation in young children. FASEB Journal. 2014;28(1)

S148. Sonneville KR, Long MW, Rifas-Shiman SL, Kleinman K, Gillman MW, Taveras EM. Associations of water and juice intake in infancy with intake of juice and sugar-sweetened beverages at 3 years of age. Obesity. 2011;19:S67-S68. doi:10.1038/oby.2011.222

S149. Te Morenga LA, Mann J, Mallard S. Dietary sugars and body weight: Systematic review and meta-analyses of randomised controlled trials. FASEB Journal. 2013;27

S150. Van Den Hooven EH, De Jonge LL, Kiefte-De Jong JC, et al. Infant macronutrient intake and cardiovascular structures and function in childhood: The generation r study. Annals of Nutrition and Metabolism. 2013;63:162. doi:10.1159/000354245

S151. Voortman T, Jaddoe V, Franco O. Macronutrient composition of early childhood diet in relation to growth and body composition. Obesity Facts. 2017;10:21. doi:10.1159/000468958

S152. Voortman T, Jaddoe V, Franco O. Macronutrient composition of early childhood diet is related to growth and adiposity during childhood. Annals of Nutrition and Metabolism. 2017;71:305. doi:10.1159/000480486

153. Voortman T, Leermakers ETM, Jaddoe VWV, et al. A posteriori and a priori dietary patterns at the age of 1 year and body composition at 6 years. European Journal of Epidemiology. 2015;30(8):951. doi:10.1007/s10654-015-0072-z
